# Transcriptomic and proteomic signatures of host NK cells delineate distinct immune states across tuberculosis infection statuses

**DOI:** 10.3389/fimmu.2025.1607770

**Published:** 2025-06-16

**Authors:** Han Zhang, Liguo Liu, Jie Hu, Xiaolin Wu, Jianhua Zheng, Henan Xin, Jiang Du, Jiarong Yang, Zizheng Lv, Zhuoran Wu, Lei Gao, Rongmei Liu, Haidan Sun, Xiaobing Zhang, Qi Jin

**Affiliations:** ^1^ National Health Commission (NHC) Key Laboratory of Systems Biology of Pathogens, State Key Laboratory of Respiratory Health and Multimorbidity, National Institute of Pathogen Biology, and Center for Tuberculosis Research, Chinese Academy of Medical Sciences and Peking Union Medical College, Beijing, China; ^2^ Department of Research Ward, Beijing Chest Hospital, Capital Medical University/Beijing Tuberculosis and Thoracic Tumor Research Institute, Beijing, China; ^3^ Institute of Basic Medical Sciences Chinese Academy of Medical Sciences, School of Basic Medicine Peking Union Medical College, Beijing, China

**Keywords:** tuberculosis, RNA sequencing, liquid chromatography-tandem mass spectrometry, natural killer cells, CD56dim NK cell, CD56bright NK cell, natural killer cell-mediated cytotoxicity, biomarker

## Abstract

**Introduction:**

Although natural killer (NK) cells play crucial roles in the immune response to *Mycobacterium tuberculosis* (*M.tb*) infection, systematic investigations delineating the immune characteristics of NK cells across the tuberculosis (TB) disease spectrum are scarce.

**Methods:**

This multiomics study employed transcriptomic, proteomic, and RT-qPCR analyses to characterize and validate CD56+ NK cells from 165 participants stratified by TB infection status (active TB (ATB), latent TB infection (LTBI), and healthy control (HC)). Peripheral blood samples from an independent cohort of 85 participants were subjected to flow cytometry analysis and validation.

**Results and discussion:**

Enrichment analyses of transcriptomic and proteomic data revealed that the NK cell-mediated cytotoxicity and apoptosis pathways were enriched in LTBI and ATB groups, whereas chemotaxis-related pathway enrichment was specific to ATB. Further analysis revealed that the expression of genes mediating the NK cell-mediated cytotoxicity signaling pathway through perforin–granzyme was upregulated in the LTBI state, whereas that of those associated with death receptors was elevated in ATB, potentially indicating a transformation of NK cell function in different TB infection states. Moreover, analysis of ATB-specific chemotaxis genes suggested that the migration of NK cells was likely to occur in the ATB state. Flow cytometry revealed an increased frequency of CD56dim NK cells and a decreased frequency of CD56bright NK cells in individuals with LTBI versus that in HCs in an independent cohort. In addition, RT-qPCR validation identified a four-biomarker combination (*SLC7A5*, *PDE4D*, *CXCR4*, and *SOCS3*) distinguishing ATB from HCs, a three-biomarker combination (*SLC7A5*, *PER1*, and *PDE4D*) differentiating LTBI from HC, and a three-biomarker combination (*SOCS3*, *GZMK*, and *HIST1H3B*) differentiating ATB from LTBI. These findings elucidate the immune clearance mechanism of NK cells in TB and provide clinically actionable biomarkers for infection staging, advancing our understanding of TB immunopathogenesis.

## 1 Introduction

Tuberculosis (TB) is an infectious disease caused by *Mycobacterium tuberculosis (M.tb)*, with a global incidence rate of 134 per 100,000 people (1). Among individuals with latent TB infection (LTBI), approximately 5–10% are at risk of progressing to active TB (ATB) (2).

Natural killer (NK) cells, as innate immune cells, play a critical role in defending against *M.tb* infection. CD56+ NK cells constitute the primary subset of NK cells in the peripheral blood (3). CD56+ NK cells are divided into two major subsets according to the differential expression of the CD56 receptor: CD56dim NK cells and CD56bright NK cells (4). The CD56dim subset accounts for more than 90% of circulating NK cells and is characterized by high expression of killer immunoglobulin-like receptors (KIRs), which are either minimally expressed or absent in the CD56bright subset. The CD56dim subset of NK cells is a highly efficient executor of cytotoxicity, containing more perforin and granzyme (5). It primarily exerts its cytotoxic effects by releasing perforin and granzyme to induce target cell death, whereas KIRs function mainly as inhibitory receptors whose interaction with HLA-C (e.g., KIR2DL1–HLA-C) effectively transmits suppressive signals (6, 7). By contrast, CD56bright NK cells are more efficient in cytokine production, mainly secreting cytokines such as interferon-γ (IFN-γ) and tumor necrosis factor-α (TNF-α). Through the death receptor pathway, which is mediated by the binding of FasL to Fas or TNF-α to TNF-R, FasL or TNF-R can induce extrinsic apoptosis, ultimately resulting in cell death (8–10). Moreover, differences in the chemotactic properties of these two NK cell subsets have become increasingly apparent. CD56bright NK cells specifically express *CCR7 (* 11), are more prevalent in secondary lymphoid organs, and tend to accumulate in inflamed tissues during pathological conditions (12).

In recent years, omics technologies have emerged as powerful tools for revealing cellular functions and regulatory networks. However, existing transcriptomic studies on TB have mainly focused on whole blood or peripheral blood mononuclear cell (PBMC) samples (13). Given the complexity of the immune response triggered by *M.tb* infection, a more detailed analysis of the functions of distinct cellular subsets is necessary. While single-cell sequencing has addressed some of these challenges, it remains limited by small sample sizes and insufficient representation. This study aimed to analyze the gene expression profiles and proteomic signatures of host CD56+ NK cells under different TB infection conditions. By integrating flow cytometry to assess changes in NK cell frequencies, this research sought to elucidate the relationships between NK cells and different TB states and the distinct molecular expression patterns associated with immune responses. These findings enhance our understanding of NK cell-mediated immune mechanisms during TB infection and provide a scientific foundation for the development of novel anti-TB strategies.

## 2 Materials and methods

### 2.1 Study design and participants

This study included a total of 250 participants: 53 ATB patients, 102 LTBI individuals, and 95 healthy controls (HCs). All ATB cases were patients diagnosed with active TB, confirmed by etiological tests, including at least one positive result from a sputum smear, *M.tb* culture, or nucleic acid amplification test. The patients were initially diagnosed with ATB and had received anti-TB treatment for less than seven days. The LTBI group was defined by a positive interferon-gamma release assay (QuantiFERON-TB Gold Plus) with no history of TB, no TB-related clinical symptoms, and no abnormal chest radiographic findings. The HC group was defined by a negative interferon-gamma release assay, no history of TB, no TB-related clinical symptoms, and no abnormal chest radiographs (14). This study was approved by the ethics committees of the Institute of Pathogen Biology, Chinese Academy of Medical Sciences (Grant No. IPB-2021-17). Informed consent was obtained from all participants prior to their inclusion in the study.

### 2.2 NK cell collection

We collected 5 mL of whole blood from each participant into K2-EDTA anticoagulant blood collection tubes. Within 4 h of collection, CD56+ cells (primarily NK cells) were isolated via the MACSxpress^®^ Whole Blood NK Cell Isolation Kit (130-127-695, Miltenyi) (15) and subsequently subjected to RNA sequencing, liquid chromatography–tandem mass spectrometry (LC-MS/MS), and RT-qPCR analyses.

### 2.3 RNA extraction and RNA sequencing

Total RNA from CD56+ NK cells was extracted following the standard protocol provided by the RNeasy Plus Mini Kit (QIAGEN). The RNA-seq libraries were prepared using the SMARTer^®^ Stranded Total RNA-Seq Kit v2 (Takara) per the manufacturer’s instructions. Quality control was performed with an Agilent 2100 Bioanalyzer, followed by sequencing on the Illumina NovaSeq 6000 platform, generating 150 bp paired-end reads. The raw data were processed via Trimmomatic for quality control and filtering (16). The filtered reads were aligned to the human reference genome (GRCh38). Transcript read counts for each sample were generated via HTSeq (17), and mRNA abundance was normalized to the number of fragments per kilobase of transcript per million mapped reads (FPKM). The Benjamini-Hochberg method was applied to calculate adjusted *P* values. Differentially expressed genes (DEGs) were analyzed via the DESeq2 algorithm to calculate fold changes (FCs), with statistical significance assessed by *P* values (18). In this study, DEGs were identified using the thresholds of *P <*0.05 and |log_2_ FC| >0.5.

### 2.4 Protein extraction and LC-MS/MS analysis

The purified CD56+ NK cells were lysed by noncontact ultrasonic treatment. The protein mixture was quantified via the BCA method. Protein digestion was subsequently conducted using a Merck Millipore PVDF membrane MultiScreen 96-well filter plate (MultiScreen HTS IP Filter Plate, 0.45 µm, clear, sterile) (19). Proteins were denatured with DTT and alkylated with IAM, followed by digestion with trypsin (50:1). The concentration of the digested peptide was measured using the Pierce Quantitative Colorimetric Peptide Assay Kit (Thermo Fisher). Mass spectrometry was performed via an Orbitrap Eclipse Tribrid mass spectrometer (Thermo Fisher Scientific) operating in positive ion mode. Data acquisition was conducted via a data-independent acquisition (DIA) method. For each sample, 800 ng of peptide was injected per run for mass spectrometry analysis, with three technical replicates per sample. A QCmix was injected after every eight samples to monitor system stability. The DIA data were analyzed in Spectronaut using the SwissProt human database with default parameters to generate the initial target list. The false discovery rate (FDR) was controlled at 1% for spectra, peptides, and proteins (20). Protein abundance values were normalized to the median protein intensity to correct for sample loading variations and systematic errors in the LC-MS/MS analysis. Differentially expressed proteins (DEPs) were identified with a threshold of *P* < 0.05.

### 2.5 Bioinformatics analysis

DEGs and DEPs were visualized using the R packages “ggplot2” for volcano plots (21) and “pheatmap” for heatmaps (22). Functional enrichment analysis was conducted via Gene Ontology (GO) and Kyoto Encyclopedia of Genes and Genomes (KEGG) pathway analyses, with the significance threshold set at *P <*0.05. The protein interaction network (PPI) was generated via STRING and Cytoscape (23).

### 2.6 Real-time quantitative PCR

cDNA was synthesized from total RNA extracted from purified CD56+ NK cells. The reactions were conducted using TaqMan™ Fast Advanced Master Mix (Thermo Fisher Scientific) and TaqMan™ Gene Expression Assays (Thermo Fisher Scientific) per the manufacturer’s instructions. Assays were performed using the ABI 7500 Fast Real-Time PCR System, with target gene expression normalized to the stably expressed reference gene *MYO1F* (assessed by our laboratory (14)). Relative expression levels were calculated via the 2^−ΔΔCt method.

### 2.7 Flow cytometry

Freshly isolated whole blood (50 µL) was treated with lysis buffer to remove red blood cells, followed by staining with various fluorochrome-conjugated antibodies. The staining panel included FITC-conjugated anti-human CD45 (555482, BD, USA), PE-conjugated anti-human CD56 (555516, BD, USA), PerCP-conjugated anti-human CD3 (552851, BD, USA), and APC-conjugated anti-human CD16 (561304, BD, USA) antibodies. After staining, the samples were washed with Cell Staining Buffer (BioLegend, 420201, USA) and immediately acquired via a BD Accuri^®^ C6 flow cytometer (BD Biosciences, USA) with CFlow Sampler software. To ensure accurate compensation and gating, irrelevant isotype and single-stained antibody controls were used to establish the compensation matrix. Single cells were identified by plotting the forward scatter height (FSC-H) against the forward scatter area (FSC-A). Gating strategies were applied according to forward/side scatter characteristics and specific fluorescence markers. Lymphocyte populations were gated using anti-CD45 staining, and non-T cells were excluded using anti-CD3 as a dump gate. NK cells were identified according to the CD3^−^CD56^+^ phenotype and further stratified into subpopulations per the fluorescence intensity of CD56 and CD16, distinguishing between CD56dim and CD56bright NK cells. Compensation adjustment and calculation of specific cell subset frequencies were performed via FlowJo Software (FlowJo LLC, USA).

### 2.8 Statistical analysis

The statistical analyses were performed with GraphPad Prism 8.0 (GraphPad Software Inc., San Diego, CA, USA). One-way ANOVA was used to analyze the differences among groups, and then the least significant difference (LSD) test was performed for multiple comparisons. All the data are presented as the means ± SD. Receiver operating characteristic (ROC) curves were generated via GraphPad Prism 8.0 to evaluate the diagnostic performance of the features selected via a LASSO regression model.

## 3 Results

### 3.1 Participant characteristics

**Figure 1:**
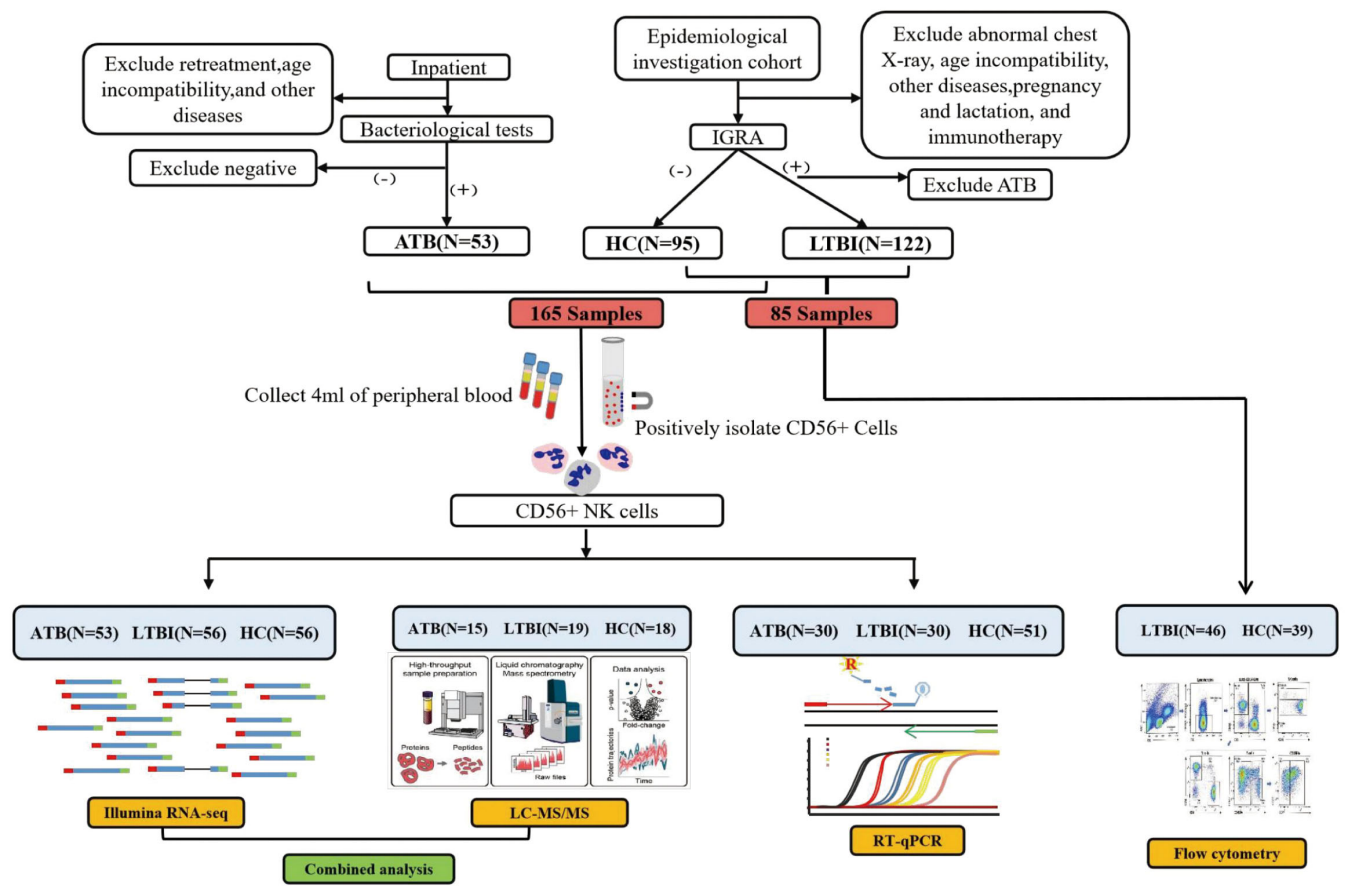
Schematic diagram of the overall study design and workflow.

This investigation included 250 participants. Among them, 165 samples, comprising those from 53 ATB patients, 56 LTBI individuals, and 56 HCs, were used for RNA-seq analysis. Among these 165 samples, 49 (from 15 ATB patients, 16 LTBI individuals, and 18 HCs) were randomly selected for LC-MS/MS analysis, whereas 111 (from 30 ATB patients, 30 LTBI individuals, and 51 HCs) were utilized for RT-qPCR analysis. Samples from another 85 individuals (46 LTBI individuals and 39 HCs) were used for flow cytometry analysis. The detailed workflow is shown in **Figure 1**. The participant characteristics are shown in **Supplementary Table S1**.

### 3.2 Transcriptomics

**Figure 2:**
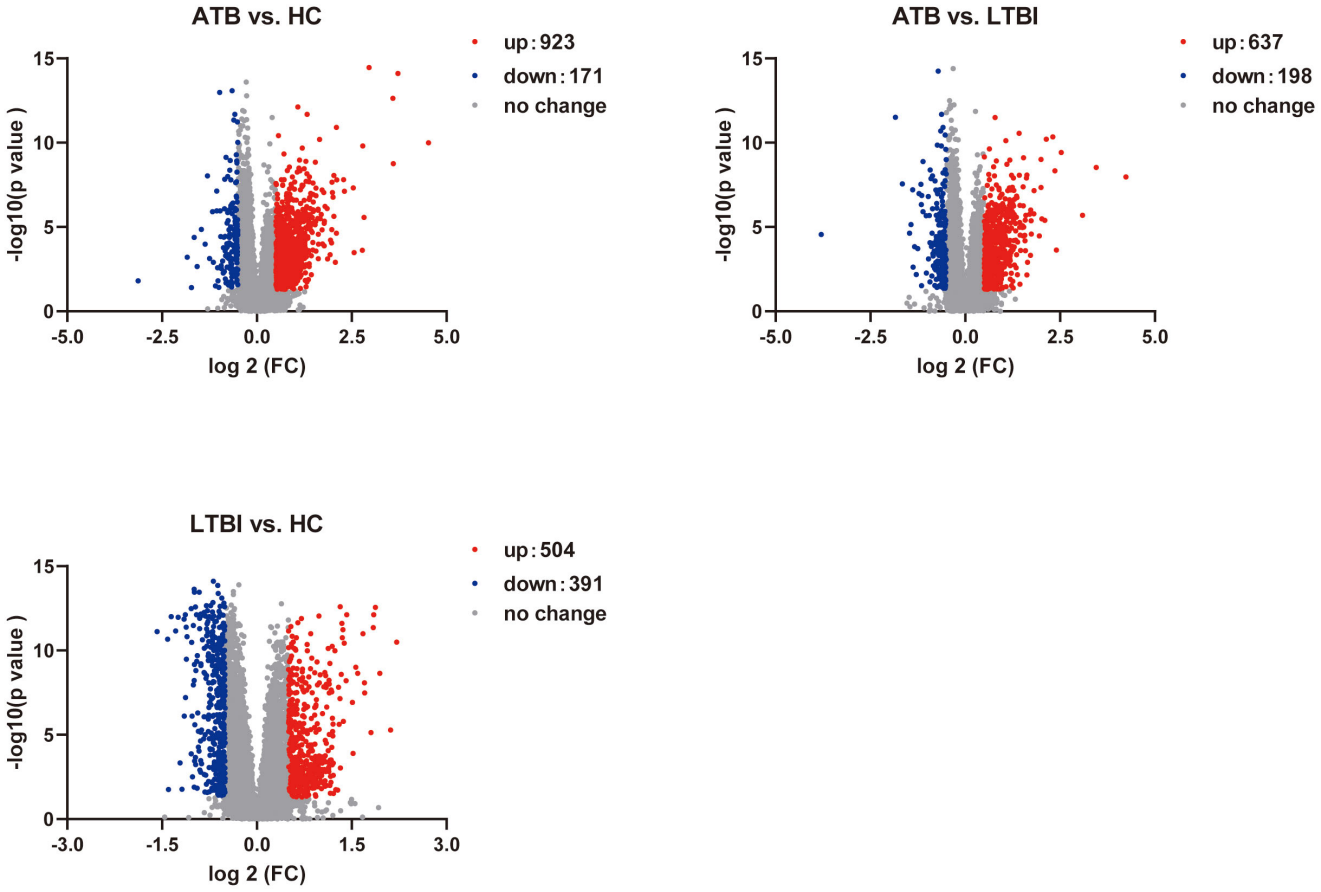
Volcano plot shows the distribution of DEGs in pairwise comparisons among the three groups of samples.

We performed RNA-seq on 165 ATB, LTBI, and HC samples to obtain mRNA expression profiles. Differential expression analysis across the three comparison groups was conducted via DESeq2. In the ATB_HC group, 1094 DEGs were identified, including 923 upregulated and 171 downregulated DEGs. In the ATB_LTBI group, 835 DEGs were identified, with 637 upregulated and 198 downregulated DEGs. For the LTBI_HC group, 895 DEGs were identified, comprising 504 upregulated and 391 downregulated DEGs. The selection criteria for DEGs were *P <*0.05 and |log_2_ FC| >0.5 (**Supplementary Table S12**). The results are presented in **Figure 2**.

#### 3.2.1 The unique transcription profile of ATB reveals chemotactic differences

**Figure 3:**
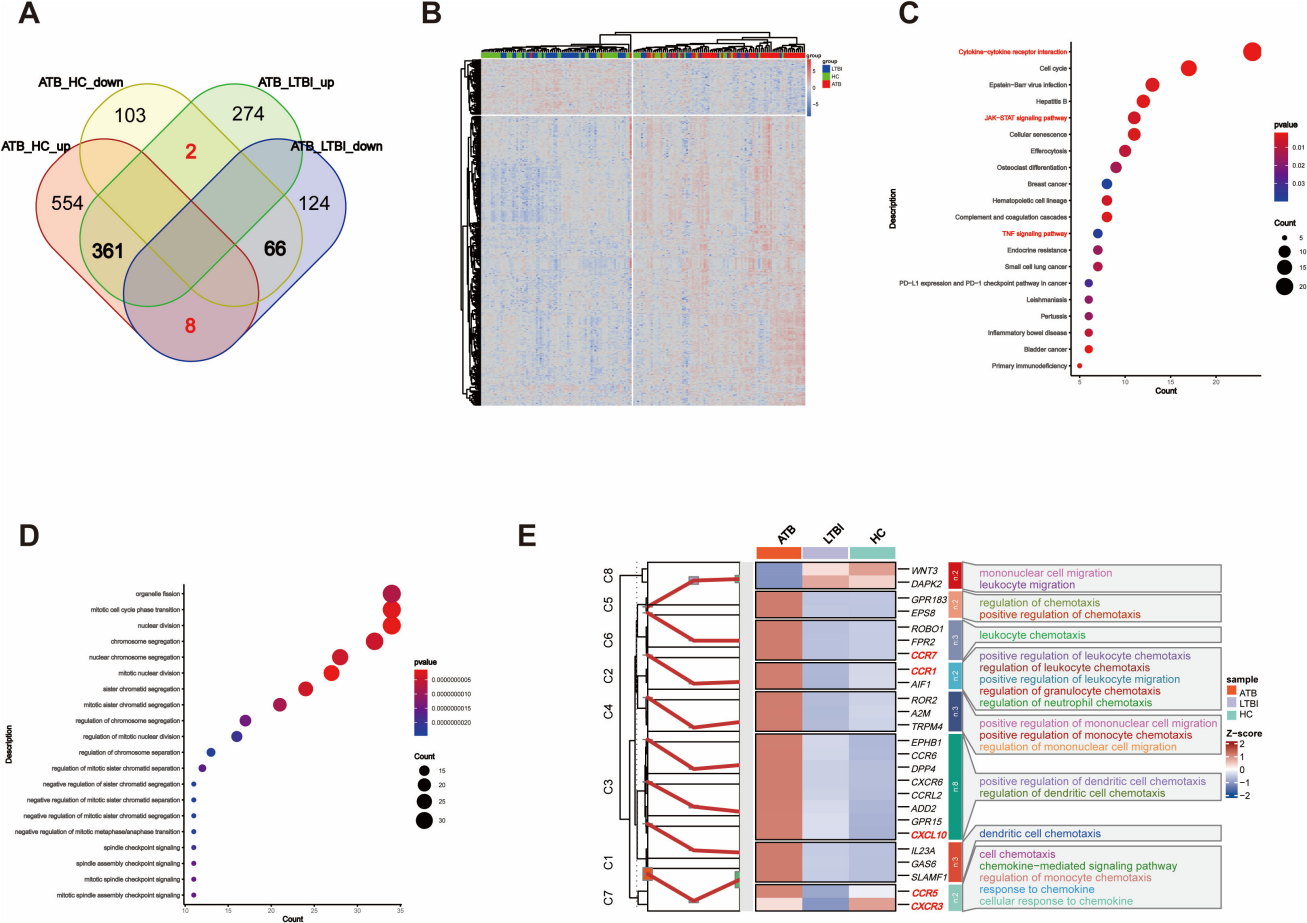
Overlap of DEGs reflects chemotaxis differences. **(A)** Venn diagram showing the overlap of DEGs between ATB_HC and ATB_LTBI. **(B)** Heatmap illustrating that the 427 overlapping DEGs are capable of distinguishing the ATB group from the LTBI and HC groups. **(C)** KEGG enrichment analysis of 427 DEGs, displaying only the top 20 most significant pathways with significant enrichment (*P <*0.05). The size of the dots corresponds to the proportion of genes annotated in each pathway. **(D)** GO enrichment analysis of 427 DEGs, displaying only the top 20 most significant pathways with significant enrichment (*P <*0.05). The size of the dots corresponds to the proportion of genes annotated in each pathway. **(E)** Display of DEGs involved in Chemotaxis-related biological processes (GO).

We first focused on ATB state-specific genes and conducted a systematic study on the expression characteristics of these genes in ATB patients and their potential biological functions. The Venn diagram shows 437 overlapping DEGs between the ATB_HC and ATB_LTBI groups. Among these DEGs, 427 exhibited consistent expression, 361 were specifically upregulated in ATB, and 66 were specifically downregulated in ATB (**Figure 3A**, **Supplementary Table S13**). Cluster analysis of these 427 ATB-specific DEGs revealed their ability to distinguish most ATB cases across different TB infection states (**Figure 3B**).

We conducted KEGG functional analysis on the 427 ATB-specific DEGs and found that the cytokine–cytokine receptor interaction pathway was the most significantly enriched (**Figure 3C**). We also identified several other functionally related pathways, such as the JAK**–**STAT, TNF, and p53 signaling pathways (**Supplementary Table S2**). Moreover, GO analysis revealed 27 terms related to immune cell differentiation, primarily involving the differentiation and differential regulation of monocytes, T cells, macrophages, and other immune cells. In addition, 45 terms related to immune function were enriched (**Figure 3D**, **Supplementary Table S3**), primarily associated with adaptive immunity and the regulation of adaptive immune responses. The enrichment of these DEGs and functional pathways suggested that, in the ATB state, NK cells modulate these genes to influence the differentiation and functional mediation of other immune cells.

Importantly, GO analysis also revealed a substantial number of terms related to chemotaxis, sparking our interest in migration across different TB infection states. Furthermore, cluster analysis was conducted on the DEGs related to chemotaxis among the different TB infection states. As shown in **Figure 3E**, of the 25 chemokine-related DEGs, all except for *WNT3* and *DAPK2* exhibited consistent changes. Compared with those in HCs, these DEGs in LTBI groups either did not significantly change or were downregulated. However, all the DEGs were upregulated in ATB group versus HC and LTBI groups. Although the difference in *CXCR3* expression was not significant, it exhibited an increasing trend (**Supplementary Table S4**).

#### 3.2.2 Opposite trend DEGs reveal functional differences in NK cells across different TB infection stages

**Figure 4:**
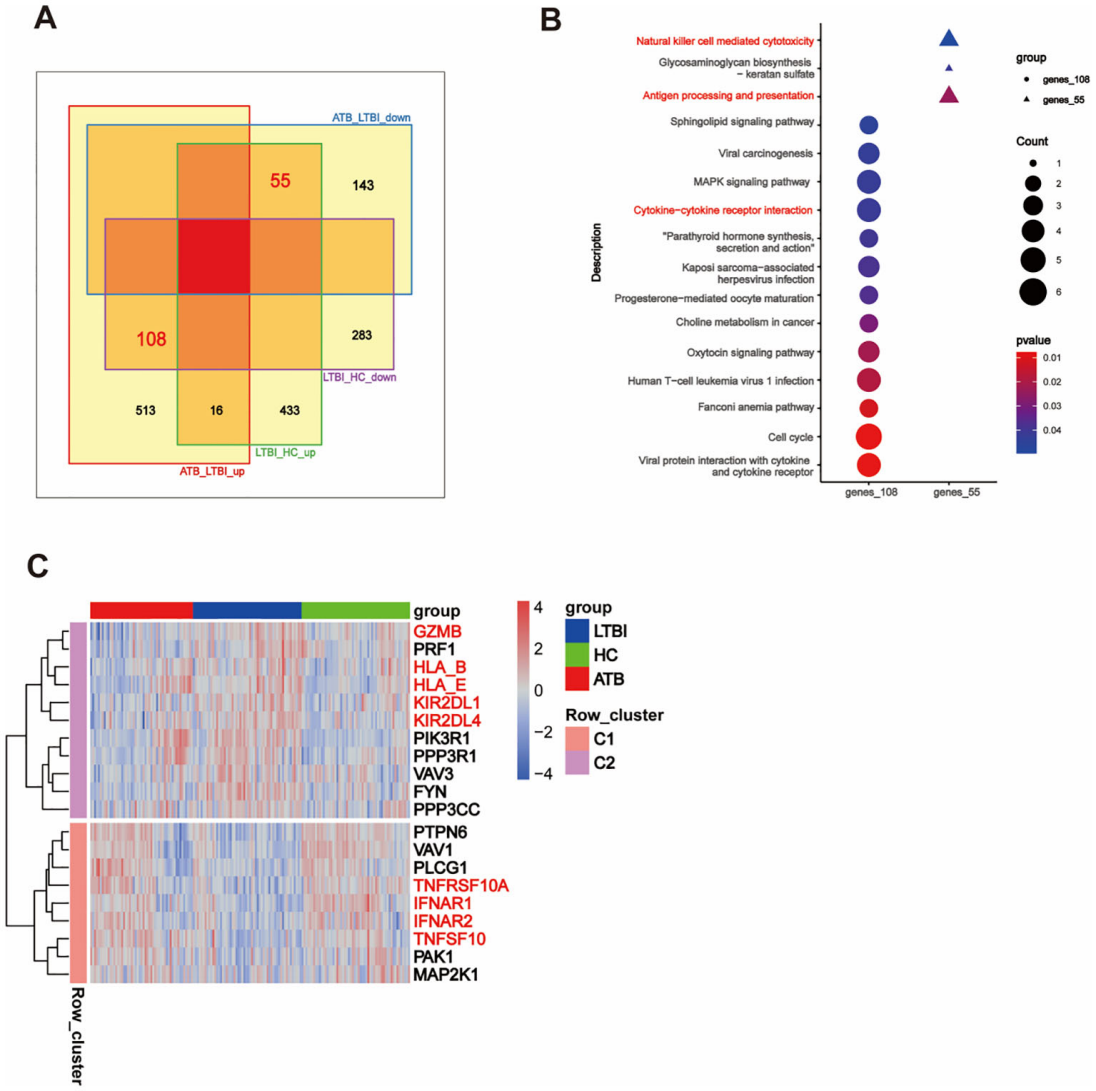
Opposite trend DEGs reveal differences in NK cell function. **(A)** Venn diagram showing the overlap of DEGs with opposite trends between ATB_LTBI and LTBI_HC groups. **(B)** KEGG enrichment analysis of 108 and 55 DEGs with opposite trends, showing significant enrichment (*P <*0.05). The size of the dots corresponds to the proportion of genes annotated in each pathway. **(C)** Heatmap illustrating the trend of DEGs in the specific Natural killer cell-mediated cytotoxicity pathway between the LTBI_HC and ATB_LTBI groups.

Furthermore, we focused on the genes with opposite expression trends between the ATB_LTBI and LTBI_HC groups. We aimed to reveal the key molecular mechanisms underlying the transition of TB infection states by analyzing which genes undergo subversive changes in different TB infection states. As shown in **Figure 4A**, 108 DEGs were upregulated in the ATB_LTBI group and downregulated in the LTBI_HC group, whereas 55 DEGs were upregulated in the LTBI_HC group and downregulated in the ATB_LTBI group. We performed KEGG functional analysis on these two sets of DEGs (**Supplementary Table S5**). Similar to the results for ATB-specific DEGs in Section 2.1, the 108 DEGs upregulated in ATB and downregulated in LTBI were enriched in the cytokine–cytokine receptor interaction pathway. Conversely, the 55 DEGs that were highly expressed in LTBI and expressed at low levels in ATB were most significantly enriched in the NK cell-mediated cytotoxicity and antigen processing and presentation signaling pathways (**Figure 4B**). Although only two DEGs, *KIR2DL1* and *KIR2DL4*, were enriched in the NK cell-mediated cytotoxicity pathway, both are specific receptors expressed in the CD56dim NK cell subset.

To further investigate the transition of the NK cell-mediated cytotoxicity pathway, we selected genes (*P <*0.05) within this pathway from both the LTBI_HC and ATB_LTBI groups. Interestingly, as shown in **Figure 4C** and **Supplementary Table S6**, 20 genes were clearly clustered into two groups across the HC, LTBI, and ATB states. Eleven genes belonged to Cluster 2 (C2), including granzyme B (*GZMB*), perforin (*PRF1*), *KIR2DL1*, *KIR2DL4*, *HLA_B*, and HLA_E, all of which were upregulated in LTBI groups and downregulated in ATB groups. Nine genes belonged to Cluster 1 (C1), including *IFNAR1*, *IFNAR2*, *TNFSF10*, and *TNFRSF10A*, which were downregulated in LTBI groups and upregulated in ATB groups. Notably, although the HLA_C gene did not exhibit the opposite expression trend in the two disease states, its expression was also upregulated in LTBI groups (**Supplementary Table S6**).

### 3.3 Proteomic validation of DEPs reveals differences in NK cells across different TB infection states

**Figure 5:**
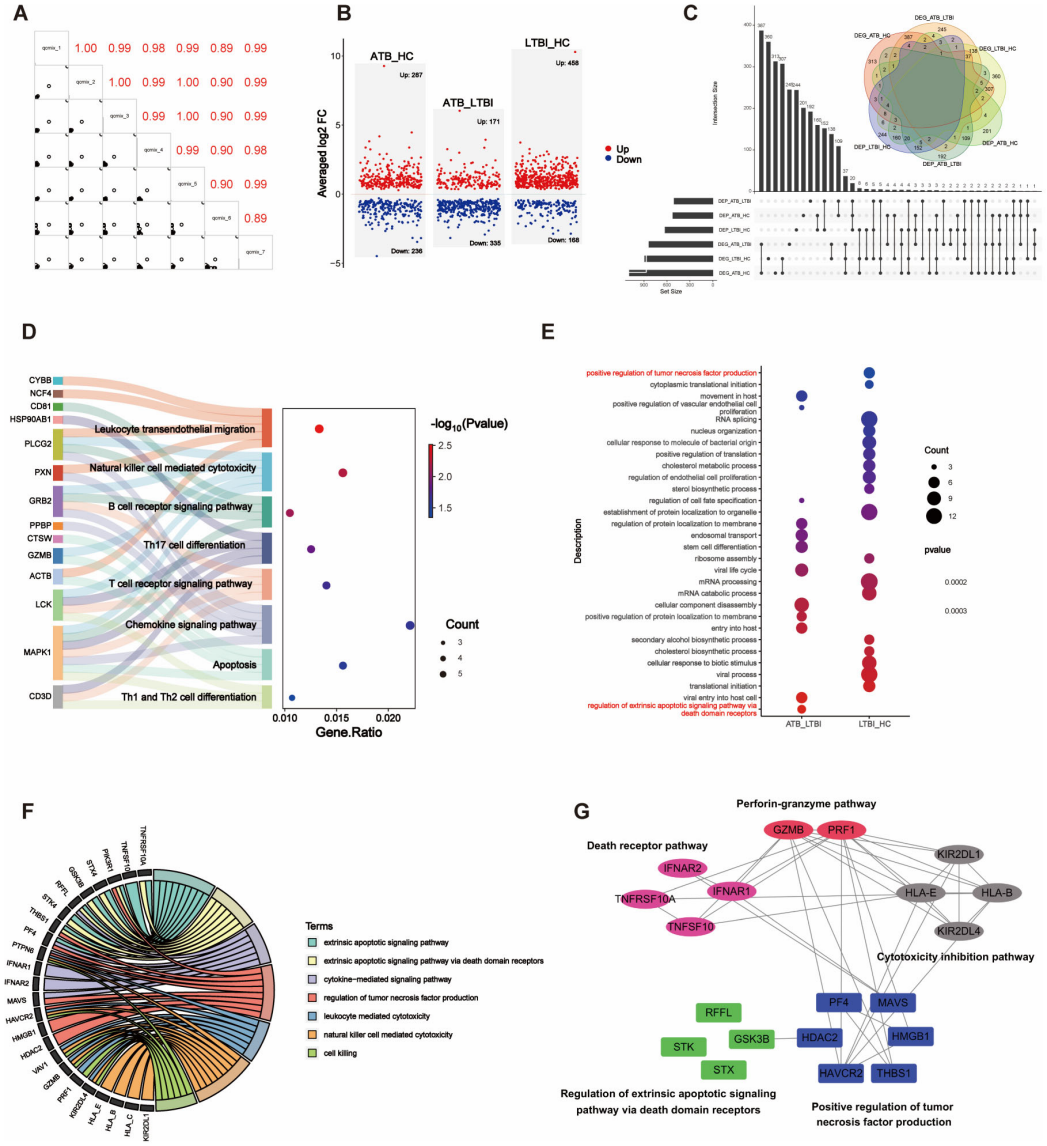
Integration analysis of DEPs and DEGs. **(A)** The correlation matrix presents the correlations in the QCmix. **(B)** Volcano plot showing the distribution of DEPs in pairwise comparisons across three groups of samples (*P <*0.05). Red represents upregulated expression, and blue represents downregulated expression. **(C)** UpSet plot and Venn diagram illustrating the overlap between DEPs and DEGs. **(D)** Sankey diagram displaying genes enriched in specific signaling pathways. **(E)** GO enrichment analysis of DEPs in the ATB_LTBI and LTBI_HC groups, with significant enrichment (*P <*0.05); only the top 20 most significant DEPs are shown. The size of the dots corresponds to the proportion of genes with corresponding annotations. **(F)** Circos plot depicting the integration of DEPs and DEGs in specific signaling pathways. **(G)** The PPI network illustrates the interaction relationships between the DEPs and DEGs involved in NK cell-mediated cytotoxicity and the apoptosis signaling pathway. The DEPs from the ATB_LTBI group are presented against a blue background; the DEPs from the LTBI_HC group are shown against a green background; the DEGs from the ATB_LTBI group are displayed against a magenta background; and the DEGs from the LTBI_HC group are depicted against pink and gray backgrounds.

We obtained proteomic profiles for 49 samples via LC-MS/MS. The QCmix correlation coefficient was greater than 0.9 (**Figure 5A**), indicating good quality control throughout the LC-MS–MS/MS process. DEPs were identified using a threshold of *P <*0.05 (**Supplementary Table S14**). As shown in **Figure 5B**, in the ATB_HC, ATB_LTBI, and LTBI_HC groups, 287, 171, and 485 upregulated and 236, 335, and 168 downregulated DEPs were observed, respectively. We integrated these DEPs with the transcriptomic profiles and revealed that 125 DEPs in the ATB_HC group, 96 in the ATB_LTBI group, and 173 in the LTBI_HC group overlapped and showed consistent expression trends with those in the transcriptome (**Figure 5C**, **Supplementary Table S7**). These DEPs were selected for further protein analysis.

First, KEGG functional enrichment analysis was performed for the DEPs of the three groups. Immune-related pathways were enriched only in the ATB_HC group (**Supplementary Table S8**). Similar to the transcriptome data, the proteomic analysis also revealed enrichment in pathways related to NK cell-mediated cytotoxicity, apoptosis, chemotaxis (such as chemokine signaling and leukocyte transendothelial migration), adaptive immunity (including B-cell receptor signaling and T-cell receptor signaling), and other immune cell differentiation pathways (such as Th17 cell differentiation and Th1/Th2 cell differentiation). These enriched pathways and the genes involved are shown in **Figure 5D**.

Second, we performed GO enrichment analysis separately on the ATB_LTBI and LTBI_HC groups. The enriched immune-related biological processes are shown in **Figure 5E**. Terms related to extrinsic apoptosis-mediated cytotoxicity, such as “regulation of extrinsic apoptotic signaling pathway via death domain receptors,” were enriched in the ATB_LTBI group. The DEPs enriched under this term included STX4, GSK3B, RFFL, and STK4. However, the term “positive regulation of tumor necrosis factor production” was enriched in the LTBI_HC group, contrasting with the transcriptome analysis results. The DEPs enriched under this term included HAVCR2, THBS1, PF4, HMGB1, MAVS, and HDAC2. We further analyzed the DEPs enriched in these two terms and the DEGs enriched in the NK cell-mediated cytotoxicity pathway in the transcriptome (genes shown in **Figure 4C**). As shown in **Figure 5F**, the abovementioned DEPs and DEGs from the ATB_LTBI group were intertwined in extrinsic apoptosis-related pathways, such as the extrinsic apoptotic signaling pathway, the extrinsic apoptotic signaling pathway via death domain receptors, and the cytokine-mediated signaling pathway. The DEPs from the LTBI_HC group, namely, HAVCR2, THBS1, PF4, HMGB1, MAVS, and HDAC2, were involved both in the regulation of biological processes such as cytotoxicity and killing (NK cell-mediated cytotoxicity, leukocyte-mediated cytotoxicity, and cell killing) and in the regulation of the biological process of tumor necrosis factor production. PPI network analysis also revealed that HAVCR2, THBS1, PF4, HMGB1, MAVS, and HDAC2 interact with these DEGs in the subpathways of NK cell-mediated cytotoxicity, including the perforin–granzyme pathway, death receptor pathway, and cytotoxicity inhibition pathway (**Figure 5G**).

### 3.4 NK cell-mediated cytotoxicity and the apoptosis signaling pathway

**Figure 6:**
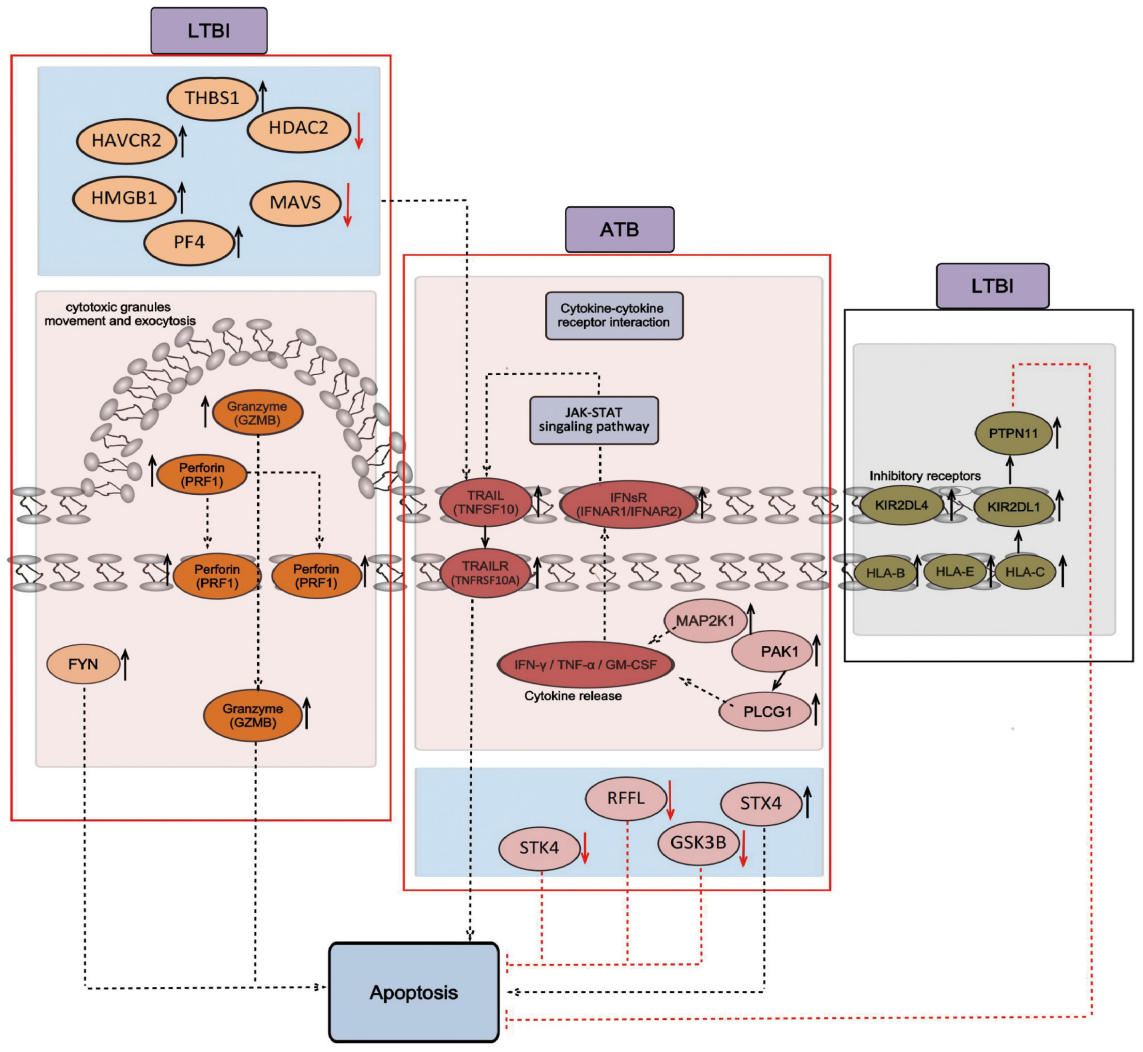
Signaling pathways of NK cell-mediated cytotoxicity and apoptosis. The black arrow indicates positive regulation, and the red inhibitory arrow indicates negative regulation. The genes obtained from RNA-seq analysis are shown on a pink background, including *GZMB*, *PRF1*, and *FYN*, which mainly mediate cytotoxicity through the perforin–granzyme pathway in the LTBI state, and *TNFSF10*, *TNFRSF10A*, *IFNR1*, and *IFNR2*, which mainly mediate cytotoxicity through the death receptor pathway in the ATB state. The proteins obtained from the LC-MS/MS analysis are shown in a blue background and include HAVCR2, THBS1, PF4, HMGB1, MAVS, and HDAC2, which regulate the production of tumor necrosis factor in the LTBI state, and STX4, GSK3B, RFFL, and STK4, which are involved in the regulation of cytotoxicity mediated by extrinsic apoptosis in the ATB state. The genes that inhibit cytotoxicity obtained from RNA-seq analysis are shown on a gray background, including *KIR2DL1*, *KIR2DL4*, *HLA_B*, *HLA_E*, *HLA_C*, and *PTPN11*, which inhibit cytotoxicity and apoptosis in the LTBI state.

The transcriptome (Section 2.2) and proteome (Section 3) KEGG analysis results revealed enrichment in the NK cell-mediated cytotoxicity signaling pathway. In the LTBI state, the expression of representative genes of the perforin–granzyme pathway (which induces intrinsic apoptosis) increased, whereas in the ATB state, the expression of representative genes of the death receptor pathway (which induces extrinsic apoptosis) increased. This effect may lead to the transformation of the NK cell-mediated cytotoxicity signaling pathway. First, genes within the perforin–granzyme pathway, such as *GZMB* and PRF1, are upregulated specifically in LTBI groups. This upregulation potentially indicates that in the LTBI state, NK cells mainly mediate cytotoxicity via the perforin–granzyme pathway. Second, increases in the expression of inhibitory receptors KIR (*KIR2DL1* and *KIR2DL4*) and HLA-I (*HLA_B*, *HLA_E*, and *HLA_C*) were also observed. This expression might suppress cytotoxicity and thereby modulate the overall immune response. In comparison, in ATB patients, the genes of the death receptor pathway, including type I interferon receptor IFNsR (*IFNAR1*, *IFNAR2*), tumor necrosis factor-α (TNF-α) family TRAIL (*TNFSF10*), and its receptor TRAILR (*TNFRSF10A*), were upregulated. This change likely leads to a shift in the NK cell-mediated cytotoxicity signaling pathway from the perforin–granzyme pathway to the death receptor pathway. Moreover, the expression of regulatory proteins such as STX4, GSK3B, RFFL, and STK4 was altered. Furthermore, in LTBI groups, HAVCR2, THBS1, PF4, HMGB1, MAVS, and HDAC2 expression was upregulated. This upregulation might be because, in the LTBI state, NK cells participate in the regulation of tumor necrosis factor production and indirectly participate in the regulation of the cytotoxicity pathway mediated by exogenous apoptosis. The specific signaling pathway diagram is shown in **Figure 6**.

### 3.5 Flow cytometry reveals frequency changes in CD56dim and CD56bright NK cell subsets in LTBI

**Figure 7:**
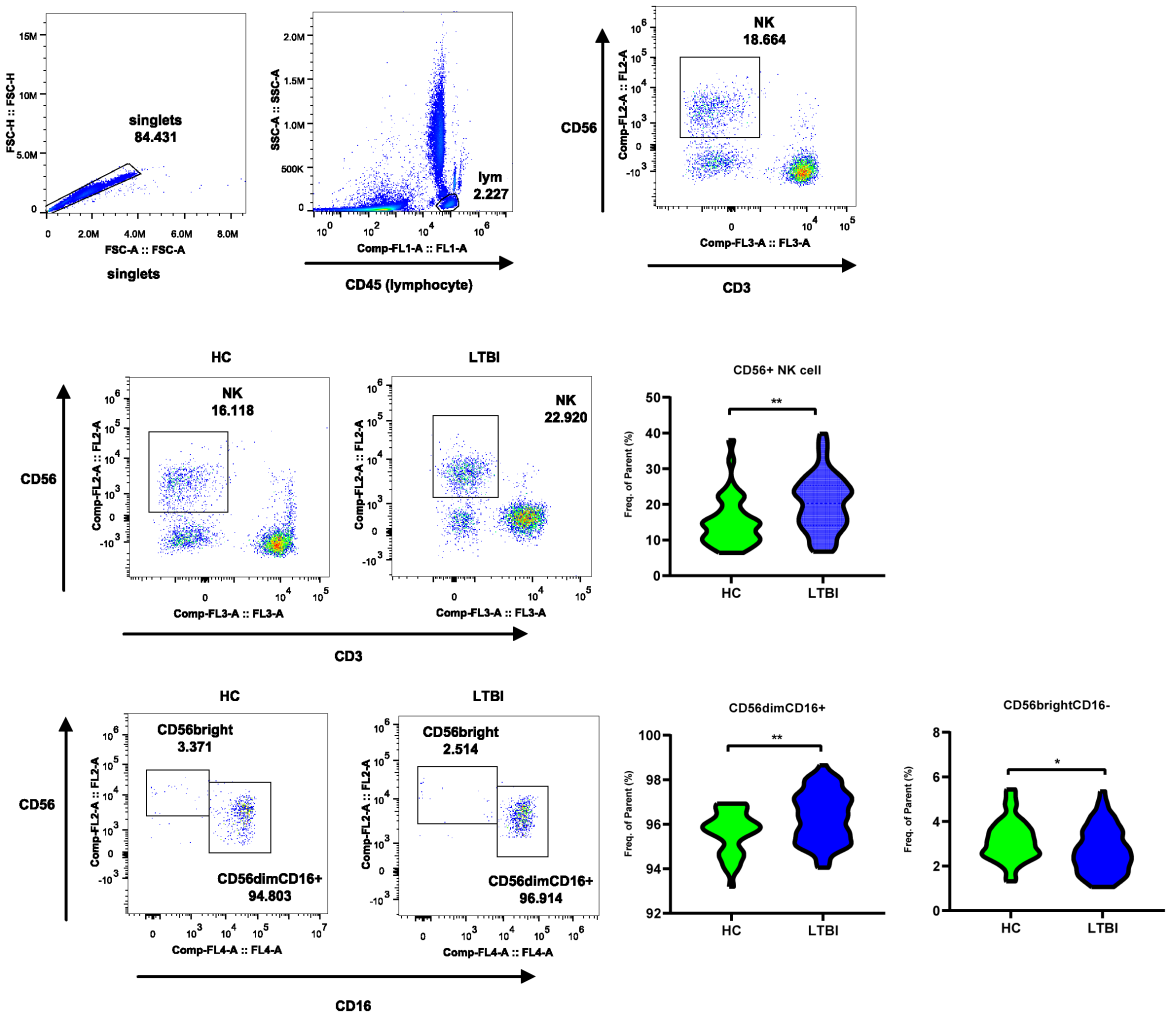
Flow cytometry reveals frequency changes in CD56dim and CD56bright NK cell subsets in LTBI. The flow cytometry plot illustrates the gating strategy for CD56+ NK Cells, CD56dim NK subset, and CD56bright subset. The violin plot shows the frequency changes of these cells between LTBI and HC groups.

To further explore the changes in the NK cell frequency and the frequencies of the two subsets in LTBI, we performed flow cytometry on an independent cohort of 85 individuals to analyze the frequency changes in CD56dim NK (primarily CD56dim CD16+ NK cells) and CD56bright NK (primarily CD56bright CD16- NK cells) cells between HCs and LTBI individuals. As shown in **Figure 7**, compared with HCs, LTBI individuals presented an increased frequency of CD56+ and CD56dim NK cells, whereas the frequency of CD56bright NK cells decreased (**Supplementary Table S15**, **Supplementary Figures S1**, **S2**).

### 3.6 RT-qPCR detection of DEGs and signature genes to distinguish ATB and LTBI from HC

**Figure 8:**
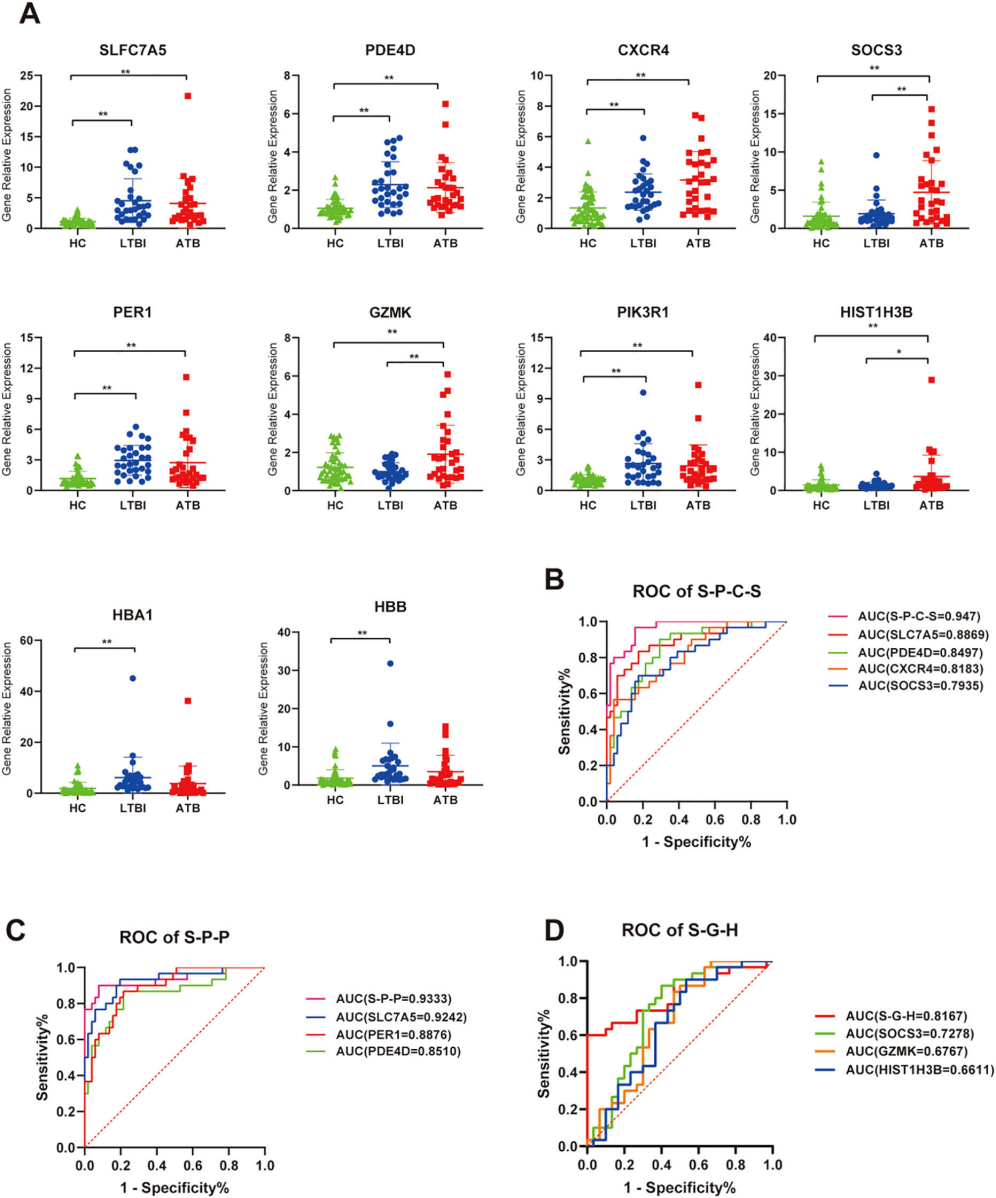
RT-qPCR validation of relative expression levels of target genes. **(A)** Scatter plot showing the relative expression of each gene across all samples. ns, *P* ≥ 0.05, *P < 0.05, **P < 0.01. **(B)** ROC curve analysis for genes combination to discriminate ATB vs HC subjects, S-P-C-S is a genes combination named by taking the initials of four genes: *SLC7A5*, *PDE4D*, *CXCR4*, and *SOCS3*. **(C)** ROC curve analysis for genes combination to discriminate LTBI vs HC subjects, S-P-P is a genes combination named by taking the initials of three genes: *SLC7A5*, *PDE4D*, and *PER1*. **(D)** ROC curve analysis for genes combination to discriminate ATB vs LTBI subjects, S-G-H is a genes combination named by taking the initials of three genes: *SOCS3*, *GZMK*, and *HIS1H3B*.

To further validate the reliability of the transcriptomic results and explore the potential of certain DEGs as auxiliary diagnostic markers for distinguishing different TB infection statuses, we performed RT-qPCR validation on 10 target genes from the RNA-seq DEGs in a validation cohort of 111 individuals (including 30 ATB patients, 30 LTBI individuals, and 51 HCs). Statistical analysis revealed that the significance and relative expression levels of genes such as *SLC7A5*, *GZMK*, *PDE4D*, *CXCR4*, *PIK3R1*, *PER1*, *SOCS3*, *HIST1H3B*, and *HBB* in the RT-qPCR validation group were consistent with the RNA-seq results. Although the RT-qPCR results for *HBA1* in the ATB_HC group did not significantly differ, the trend was consistent with the RNA-seq findings (**Figure 8A**). In addition, we further utilized a LASSO regression model to combine the validated genes and constructed receiver operating characteristic (ROC) curves to distinguish between the phenotypes (ATB, LTBI, and HC). In the ATB_HC comparison, a combination of four genes—*SLC7A5*, *PDE4D*, *CXCR4*, and *SOCS3*—was the most effective for distinguishing the ATB and HC groups, with an AUC of 0.947 (**Figure 8B**). When the Youden index was maximized, the sensitivity was 0.867, and the specificity was 0.882 (**Supplementary Table S9**). In the LTBI_HC comparison, a combination of three genes—*SLC7A5*, *PER1*, and *PDE4D*—was the most effective for distinguishing the LTBI and HC groups, with an AUC of 0.933 (**Figure 8C**). At the optimal Youden index, the sensitivity was 0.900, and the specificity was 0.922 (**Supplementary Table S10**). In the comparison between ATB and LTBI, a combination of three genes—*SOCS3*, *GZMK*, and *HIST1H3B*—was the most effective in differentiating the ATB and LTBI groups, with an AUC of 0.817 (**Figure 8D**). At the optimal Youden index, the sensitivity was 0.633, and the specificity was 0.900 (**Supplementary Table S11**). These gene combinations demonstrated strong discriminatory power in distinguishing different TB infection states; thus, they are promising potential diagnostic biomarkers.

## 4 Discussion

In this study, we aimed to elucidate the immune mechanisms and functional characteristics of peripheral blood NK cells across different infection states of TB by analyzing the differences in the transcriptional and protein profiles of NK cells in the peripheral blood of ATB, LTBI and HC individuals. Initially, we explored DEGs strongly associated with ATB and identified significant changes in a wide range of cytokines and cytokine receptors. These changes mediate mainly cytokine–cytokine receptor interaction signaling pathways. GO enrichment analysis revealed numerous DEGs involved in adaptive immune cell differentiation, immune function, and regulation. Proteomic analysis also revealed enrichment of adaptive immune-related pathways (B-cell receptor signaling pathway and T-cell receptor signaling pathway) in the ATB_HC group and other immune cell differentiation pathways (e.g., Th17 cell differentiation and Th1 and Th2 cell differentiation). These findings suggest that when ATB is present, NK cells indirectly participate in adaptive immunity by regulating the differentiation and function of other immune cells, a phenomenon previously reported in TB research (24).

Importantly, both transcriptomic and proteomic analyses identified chemotaxis-related terms, highlighting numerous chemotactic genes significantly upregulated in ATB. IP-10 (interferon-inducible protein 10), encoded by the *CXCL10* gene, is prominently featured in the NK chemotactic gene set and is crucial in stimulating NK cell migration. CXCR3+ NK cells are the primary targets of IP-10 (25). In this study, both IP-10 and *CXCR3* were elevated in the NK cells of ATB patients, suggesting that in ATB patients, NK cells exhibit a heightened response to chemotactic signals, with increased expression of surface receptors that facilitate their migration to the site of infection. Previous studies have demonstrated that supernatant from *M.tb*-infected dendritic cells stimulates NK cell migration and increases *CXCR3* expression, with chemotactic activity primarily attributed to *CXCL10 (* 26). The chemokine receptor *CCR7*, specific to CD56bright NK cells, was highly expressed in the NK cells of ATB patients. These findings suggest that CD56bright NK cells undergo migration in ATB. Elevated expression of *CCR7* in the CD56bright subset has been reported in TB pleuritis (27). Furthermore, the chemokine receptors *CCR1* and *CCR5* are considered characteristic chemokine receptors for NK-macrophage crosstalk and migration (28). In this study, these receptors were highly expressed in NK cells from ATB patients, suggesting that in ATB patients, NK cells and macrophages may migrate in coordination.

In addition, numerous other chemokines, chemokine receptors, and genes regulating chemotaxis showed the same expression pattern, with their levels being unchanged or decreased in NK cells of LTBI groups and increased in those of ATB patients. This finding reflects a relatively stable immune equilibrium in the LTBI state. However, in the ATB state, the inflammatory response intensifies, with a surge of inflammatory mediators and chemokines providing strong migratory signals that could guide NK cells to migrate from the bloodstream or peripheral tissues to the site of infection (27). This result also helps explain previous studies demonstrating NK cell subset depletion in the PBMCs of ATB patients (29). Similarly, evidence of the regulation of chemotaxis by NK cells was found in our proteomic analysis. Notably, chemokines have complex functions beyond simply guiding cell migration. Elevated levels of chemokines and their receptors can also promote the activation of immune cells, enhancing their phagocytic and bactericidal abilities against *M.tb.*

Furthermore, by analyzing genes exhibiting opposite expression patterns between the two TB infection states, we explored the shift in NK cells’ immune mechanisms during distinct TB infection states. The characteristic genes mediating the NK cell-mediated cytotoxicity signaling pathway through perforin–granzyme were upregulated in the LTBI state, whereas those via death receptors were elevated in ATB. Specifically, in the LTBI state, genes encoding perforin (*PRF1*), granzyme B (*GZMB*), and the gene *FYN (* 30), which promotes perforin release, as well as inhibitory KIR receptors (*KIR2DL1* and *KIR2DL4*) and HLA molecules (*HLA-B*, *HLA-C*, and *HLA-E*), were highly expressed. These genes are characteristic of the CD56dim NK cell subset, suggesting that in LTBI, cytotoxic effects mediated primarily by perforin and granzyme are crucial for the early control of *M.tb* infection. Consistent with the gene characteristics, through flow cytometry, we analyzed the frequency changes in CD56 NK cell subsets in the peripheral blood of individuals with LTBI and found that, compared with those in the HCs, the proportions of CD56+ NK cells and the CD56dim NK cell subset were significantly greater in the LTBI individuals, whereas the proportion of the CD56bright NK cell subset was lower. The greater proportion of the CD56dim subset, owing to its stronger cytotoxic function than the CD56bright subset (5), can control the progression of TB. This subset of NK cells also expresses high levels of inhibitory receptors and HLA molecules, which may suppress toxic effects. Previous studies have shown that *KIR2DL1* recognizes and binds to the ligand *HLA-C* to mediate cytotoxic inhibition (7, 31) and that excessive cytotoxicity can accelerate the formation of pulmonary TB cavities (32). However, in the ATB state, we observed a decrease in the expression of genes involved in the cytotoxicity mediated by perforin and granzyme. Conversely, the expression of genes associated with death receptor-mediated cytotoxicity, which are also genes specifically and highly expressed in the CD56bright subset, such as type I interferon receptors (*IFNAR1* and *IFNAR2*), the TNF-α family member TRAIL (*TNFSF10*), and its receptor TRAILR (*TNFRSF10A*), was upregulated. This expression upregulation might have resulted in a shift in cytotoxicity toward extrinsic apoptosis mediated by death receptors, which may be driven primarily by the CD56bright NK cell subset (6, 33). We did not investigate changes in the CD56 NK cell subsets of patients with ATB. The frequency changes in NK cell subsets in ATB patients are affected by multiple factors, such as the host’s pathological state, immune environment, and chemotaxis. Prior studies have also shown that, compared with LTBI, ATB is associated with a decrease in early NK cells (CD16+ CD56dim CD57+) and an increase in the proportion of the CD56bright CD16dim NK cell subset (29, 34). Similarly, proteomic analysis revealed enrichment of the “NK cell-mediated cytotoxicity” and “apoptosis” pathways in the ATB_HC comparison, providing consistent evidence with the transcriptomic data. We found that the levels of some proteins regulating the extrinsic apoptotic signaling pathway were elevated in the NK cells of patients with ATB. Among them, some are proapoptotic (GSK3B and STK4) (35, 36), whereas others are antiapoptotic (STX4 and RFFL) (37, 38). Furthermore, through proteomic analysis, we found that the levels of proteins involved in the regulation of tumor necrosis factor production, such as HAVCR2, THBS1, PF4, HMGB1, MAVS, and HDAC2, were elevated in the NK cells of individuals with LTBI. All of these proteins are conducive to the production of tumor necrosis factor (39–44). These findings imply that, in the LTBI state, NK cells may regulate the production of tumor necrosis factor via these proteins, thereby participating in the cytotoxic process mediated by exogenous apoptosis. These proteins collectively regulate the “NK cell-mediated cytotoxicity” and “apoptosis” pathways, forming a complex dynamic regulatory network. Additionally, through the integrated analysis of transcriptomics and proteomics, we observed divergence between the two omics results. The divergence may be attributed to post-transcriptional regulatory mechanisms (such as mRNA stability, translation efficiency, and protein degradation) (45–47). Such divergence provided insights for future screening of biomarkers and validation of therapeutic targets: sole reliance on transcriptomic data may result in the omission of key functional proteins, whereas integrative analysis of multi-omics data can more comprehensively characterize biological processes and significantly enhance biomarker reliability (48). Therefore, in clinical translational research, potential targets predicted by transcriptomics should be interpreted with caution, and systematic validation using multi-omics data is recommended to minimize false-positive risks.

In addition, TB diagnosis has long been a challenge. While bacteriological confirmation remains the gold standard, diagnostic rates have been consistently low, with little improvement observed. Host transcriptomics can reflect the immune response status, which helps elucidate the pathogenesis and progression of TB and holds significant potential for enhancing clinical diagnostics. Currently, TB remains devoid of universally acknowledged clinical biomarkers, with the core challenge lying in the difficulty of replicating biomarkers across large-scale populations. Notable disparities in genetic backgrounds and environmental exposures among populations may give rise to substantial heterogeneity in gene/protein expression profiles across different ethnicities and regions (49, 50), which could further compromise the reproducibility of biomarkers in cross-ethnic and cross-regional cohorts (51). Most ongoing TB biomarker investigations focus on whole blood or PBMC samples (13), yet the complexity and cellular heterogeneity of these specimens may obscure critical biological signals. By contrast, the exploration of biomarkers based on NK cells as a single immune cell population is anticipated to enhance biomarker specificity and stability, thereby offering a novel research perspective for addressing the reproducibility challenges of TB biomarkers. Based on peripheral blood NK cells, we used RT-qPCR to validate and identify a four-gene combination (*SLC7A5*, *PDE4D*, *CXCR4*, and *SOCS3*) that distinguishes ATB patients from HCs and a three-gene combination (*SLC7A5*, *PER1*, and *PDE4D*) that differentiates LTBI from HC, showing considerable diagnostic potential in terms of sensitivity and specificity. In addition, the three-gene combination (*SOCS3*, *GZMK*, and *HIST1H3B*) exhibited suboptimal sensitivity in differentiating ATB from LTBI, limiting its utility as a standalone screening tool. However, its high specificity supports its potential as an adjunct to clinical diagnosis, particularly in reducing false-negative results for LTBI. Meanwhile, we observed that certain biomarkers (*SLC7A5*, *PDE4D*, *CXCR4*, *PER1*, *PIK3R1*, *HBA1*, *HBB*) were elevated during the pre-infection phase (LTBI), while others (*SOCS3*, *GZMK*, *HIS1H3B*) were upregulated in the post-infection phase (ATB). This phenomenon suggests the potential value of biomarkers in tracking tuberculosis progression. Additionally, previous studies have demonstrated that immune markers such as GZMB and soluble TRAILR can predict treatment outcomes during the intensive phase of tuberculosis (52), which also reflects the potential of the characteristic genes identified in this study for monitoring disease progression or treatment response. Of course, more in-depth studies incorporating samples from different disease development stages will be required to clarify their clinical value. Notably, the potential biomarkers identified in this study are still in the preliminary stage and remain far from clinical application. Their stability and generalizability across different ethnic and regional populations still need to be explored. Future research will expand the sample size to explore and validate robust diagnostic biomarkers (particularly for differentiating ATB from LTBI) to optimize the model.

This study has some limitations. In terms of sample size, there is an urgent need to expand the current cohort by including more participants representing diverse subgroups and infection stages. This strategy would not only enhance the accuracy and generalizability of existing diagnostic models but, when combined with dynamic tracking of disease progression data, could also provide key insights into the immunological mechanisms underlying TB pathogenesis, thereby revealing disease evolution patterns and potential therapeutic targets. Additionally, functional validation requires further strengthening: by integrating *in vitro* cell models, animal infection experiments, and emerging spatial transcriptomics technologies [e.g., spatial CITE-seq (53), multimodal tri-omics mapping (54), and Perturb-DBiT (55, 56)], it is expected to systematically construct the molecular regulatory network of NK cells, deepen mechanistic interpretation, and address the shortcomings of traditional omics technologies in resolving cellular microenvironments.

In conclusion, this study systematically characterized the molecular and functional signatures of peripheral blood NK cells across ATB, LTBI, and HC states. In LTBI, NK cells exhibit upregulated perforin–granzyme-mediated cytotoxicity pathways, increased inhibitory receptor and HLA ligand expression, and a higher frequency of CD56dim subsets, suggesting a balanced immune state dominated by intrinsic cytotoxicity with self-regulation. By contrast, ATB is characterized by the activation of death receptor-mediated extrinsic apoptosis pathways and the significant upregulation of chemokines/chemokine receptors, which may promote NK cell migration. Flow cytometry confirmed that LTBI is associated with elevated CD56dim and reduced CD56bright NK cell frequencies compared with those observed in HCs. RT-qPCR was used to validate multigene biomarker panels with high diagnostic accuracy for distinguishing ATB and LTBI from HC. The observed differences in the NK cell profiles between LTBI individuals and ATB patients could reflect dynamic regulatory mechanisms, possibly supporting the role of immune control in latency and migratory responses in active disease.

## Data Availability

The data presented in the study are deposited in the National Genomics Data Center (NGDC), accession number PRJCA040900.
