# Supplementary material for: Transcriptomic and proteomic signatures of host NK cells delineate distinct immune states across tuberculosis infection statuses
Source: Front Immunol. 2025 Jun 16;16:1607770. doi: 10.3389/fimmu.2025.1607770 (PMC12206628; doi:10.3389/fimmu.2025.1607770)
Supplement: Supplementary file 1 [file DataSheet1.pdf]

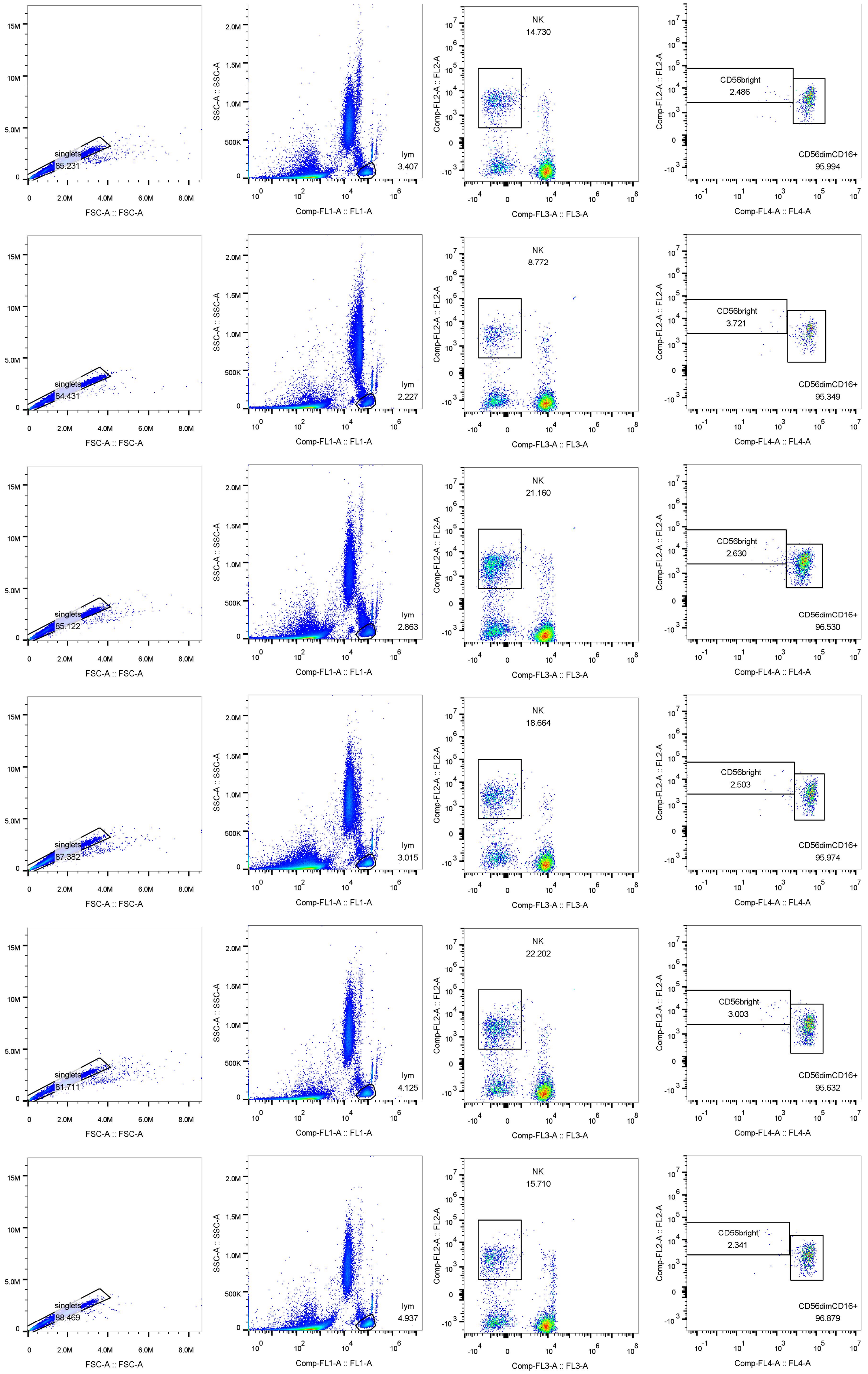
singlets
85.231
FSC-A :: FSC-A
SSC-A :: SSC-A
lym
3.407
Comp-FL1-A :: FL1-A
NK
14.730
Comp-FL2-A :: FL2-A
Comp-FL3-A :: FL3-A
CD56bright
2.486
CD56dimCD16+
95.994
Comp-FL4-A :: FL4-A
singlets
84.431
lym
2.227
NK
8.772
CD56bright
3.721
CD56dimCD16+
95.349
singlets
85.122
lym
2.863
NK
21.160
CD56bright
2.630
CD56dimCD16+
96.530
singlets
87.382
lym
3.015
NK
18.664
CD56bright
2.503
CD56dimCD16+
95.974
singlets
81.711
lym
4.125
NK
22.202
CD56bright
3.003
CD56dimCD16+
95.632
singlets
88.469
lym
4.937
NK
15.710
CD56bright
2.341
CD56dimCD16+
96.879

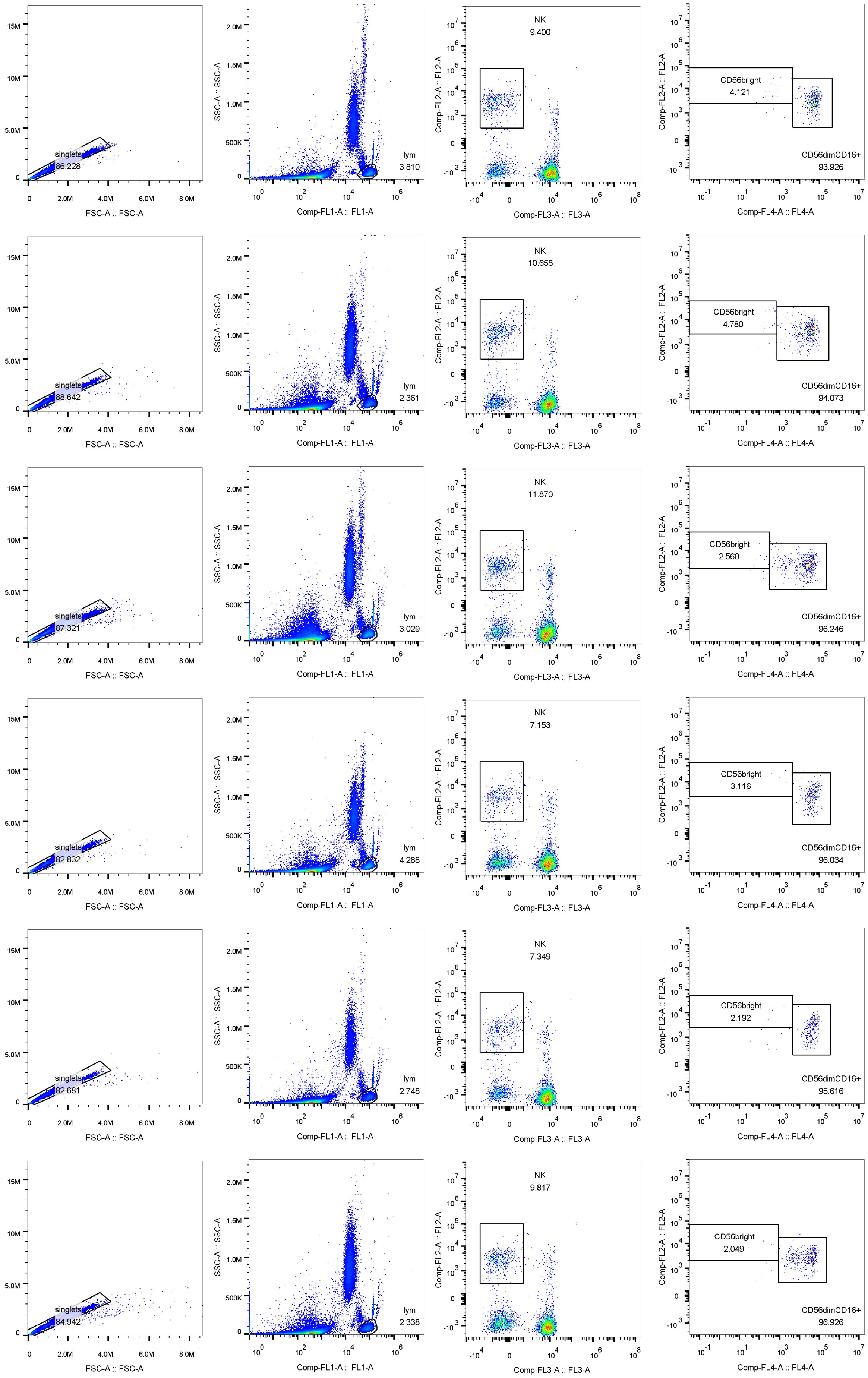
singlets
86.228
FSC-A :: FSC-A
SSC-A :: SSC-A
lym
3.810
Comp-FL1-A :: FL1-A
NK
9.400
Comp-FL2-A :: FL2-A
Comp-FL3-A :: FL3-A
CD56bright
4.121
CD56dimCD16+
93.926
Comp-FL4-A :: FL4-A
singlets
88.642
lym
2.361
NK
10.658
CD56bright
4.780
CD56dimCD16+
94.073
singlets
87.321
lym
3.029
NK
11.870
CD56bright
2.560
CD56dimCD16+
96.246
singlets
82.832
lym
4.288
NK
7.153
CD56bright
3.116
CD56dimCD16+
96.034
singlets
82.681
lym
2.748
NK
7.349
CD56bright
2.192
CD56dimCD16+
95.616
singlets
84.942
lym
2.338
NK
9.817
CD56bright
2.049
CD56dimCD16+
96.926

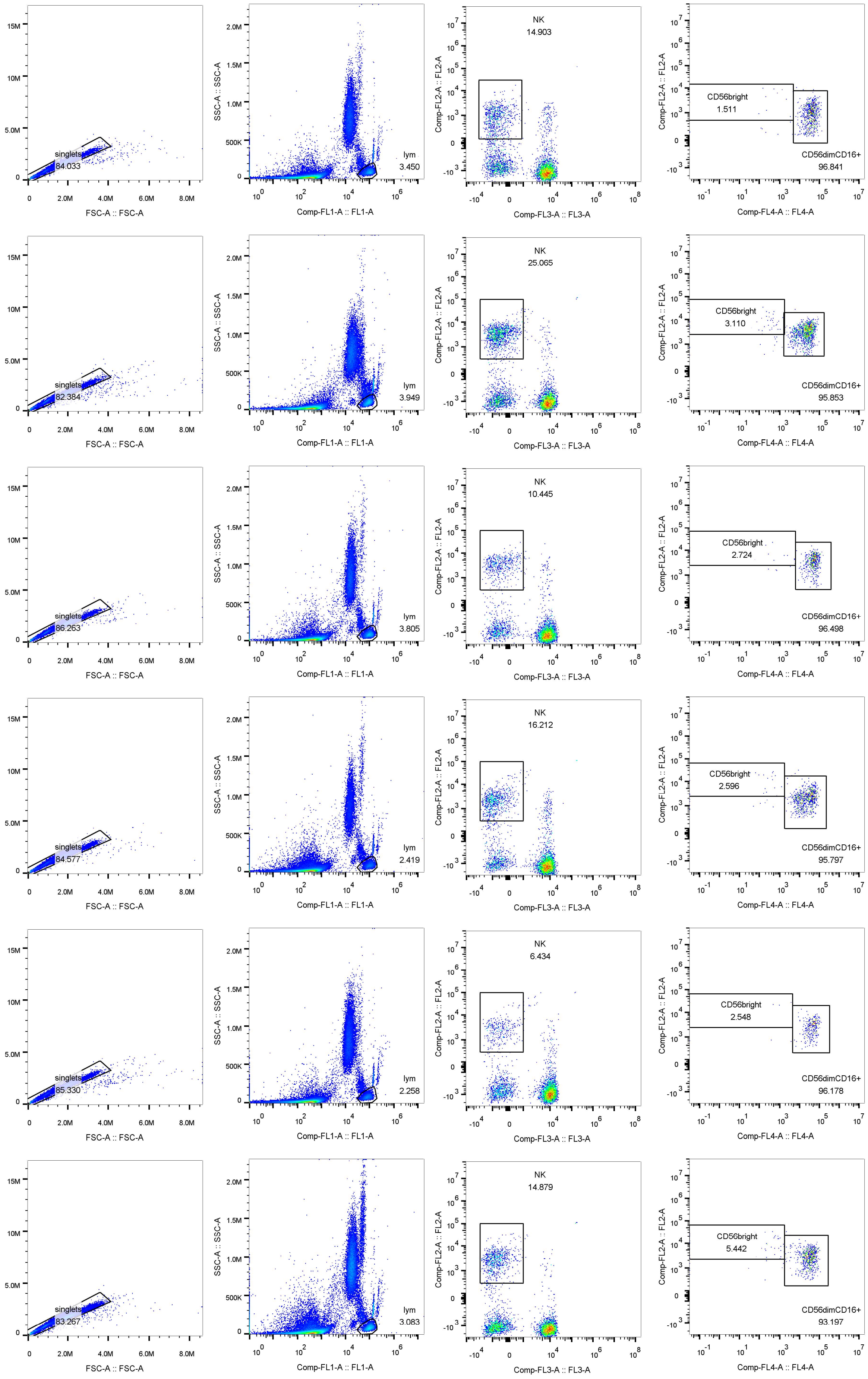

singlets
84.033
FSC-A :: FSC-A
SSC-A :: SSC-A
lym
3.450
Comp-FL1-A :: FL1-A
NK
14.903
Comp-FL2-A :: FL2-A
Comp-FL3-A :: FL3-A
CD56bright
1.511
CD56dimCD16+
96.841
Comp-FL4-A :: FL4-A
singlets
82.384
lym
3.949
NK
25.065
CD56bright
3.110
CD56dimCD16+
95.853
singlets
86.263
lym
3.805
NK
10.445
CD56bright
2.724
CD56dimCD16+
96.498
singlets
84.577
lym
2.419
NK
16.212
CD56bright
2.596
CD56dimCD16+
95.797
singlets
85.330
lym
2.258
NK
6.434
CD56bright
2.548
CD56dimCD16+
96.178
singlets
83.267
lym
3.083
NK
14.879
CD56bright
5.442
CD56dimCD16+
93.197

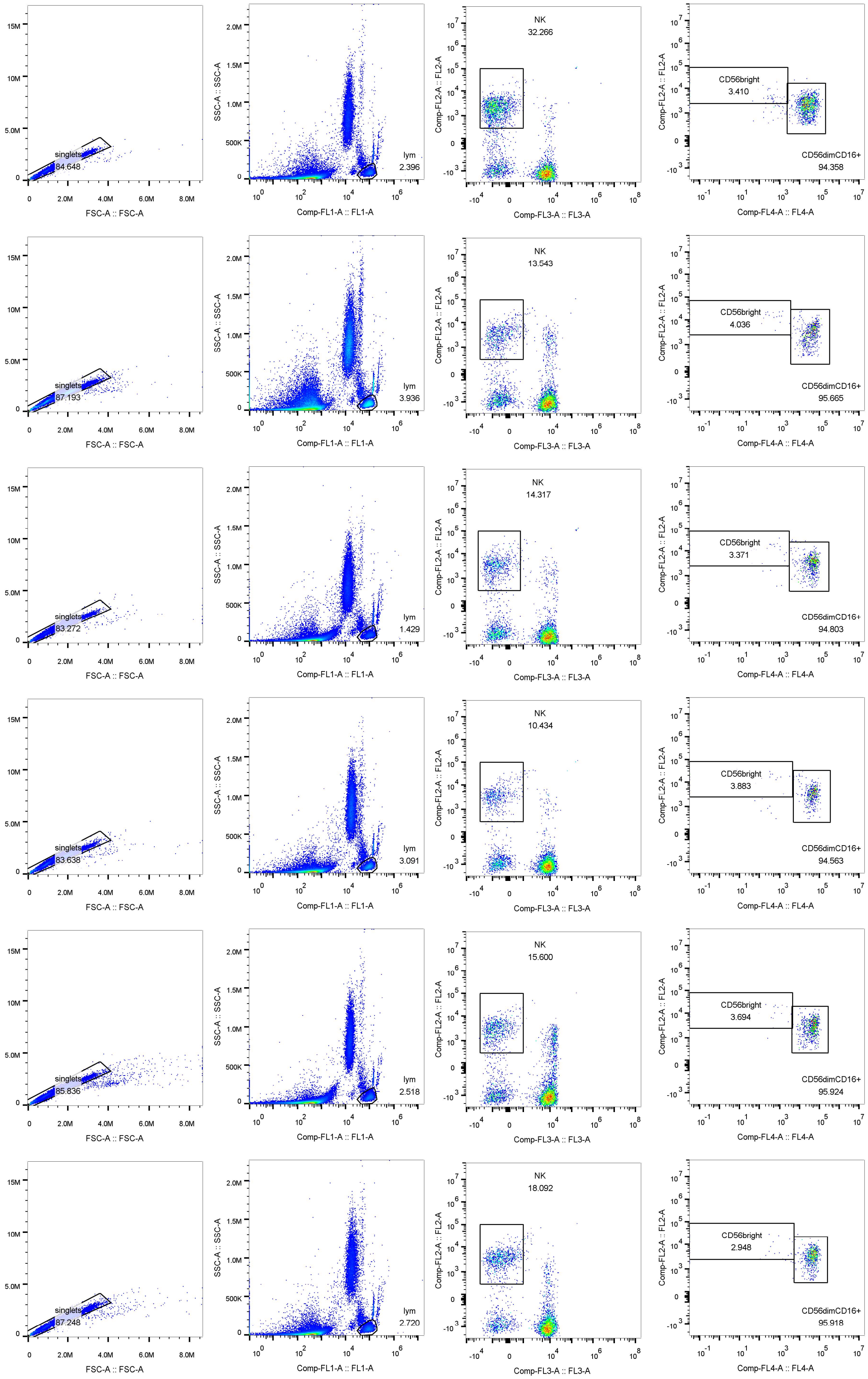
FSC-A :: FSC-A
SSC-A :: SSC-A
Comp-FL1-A :: FL1-A
Comp-FL2-A :: FL2-A
Comp-FL3-A :: FL3-A
Comp-FL4-A :: FL4-A
singlets
84.648
lym
2.396
NK
32.266
CD56bright
3.410
CD56dimCD16+
94.358
singlets
87.193
lym
3.936
NK
13.543
CD56bright
4.036
CD56dimCD16+
95.665
singlets
83.272
lym
1.429
NK
14.317
CD56bright
3.371
CD56dimCD16+
94.803
singlets
83.638
lym
3.091
NK
10.434
CD56bright
3.883
CD56dimCD16+
94.563
singlets
85.836
lym
2.518
NK
15.600
CD56bright
3.694
CD56dimCD16+
95.924
singlets
87.248
lym
2.720
NK
18.092
CD56bright
2.948
CD56dimCD16+
95.918

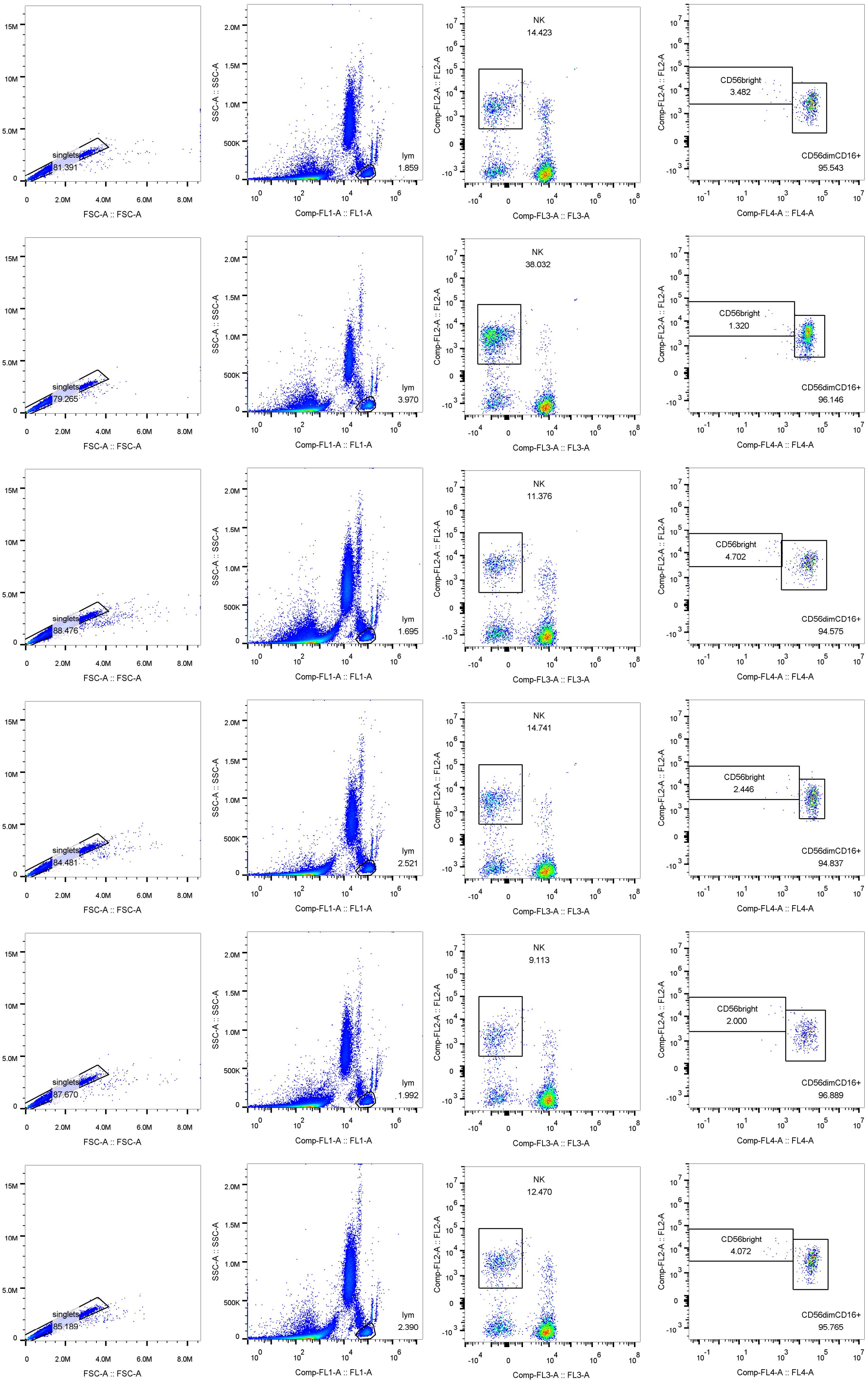
FSC-A :: FSC-A
SSC-A :: SSC-A
Comp-FL1-A :: FL1-A
Comp-FL2-A :: FL2-A
Comp-FL3-A :: FL3-A
Comp-FL4-A :: FL4-A
singlets
81.391
lym
1.859
NK
14.423
CD56bright
3.482
CD56dimCD16+
95.543
singlets
79.265
lym
3.970
NK
38.032
CD56bright
1.320
CD56dimCD16+
96.146
singlets
88.476
lym
1.695
NK
11.376
CD56bright
4.702
CD56dimCD16+
94.575
singlets
84.481
lym
2.521
NK
14.741
CD56bright
2.446
CD56dimCD16+
94.837
singlets
87.670
lym
1.992
NK
9.113
CD56bright
2.000
CD56dimCD16+
96.889
singlets
85.189
lym
2.390
NK
12.470
CD56bright
4.072
CD56dimCD16+
95.765

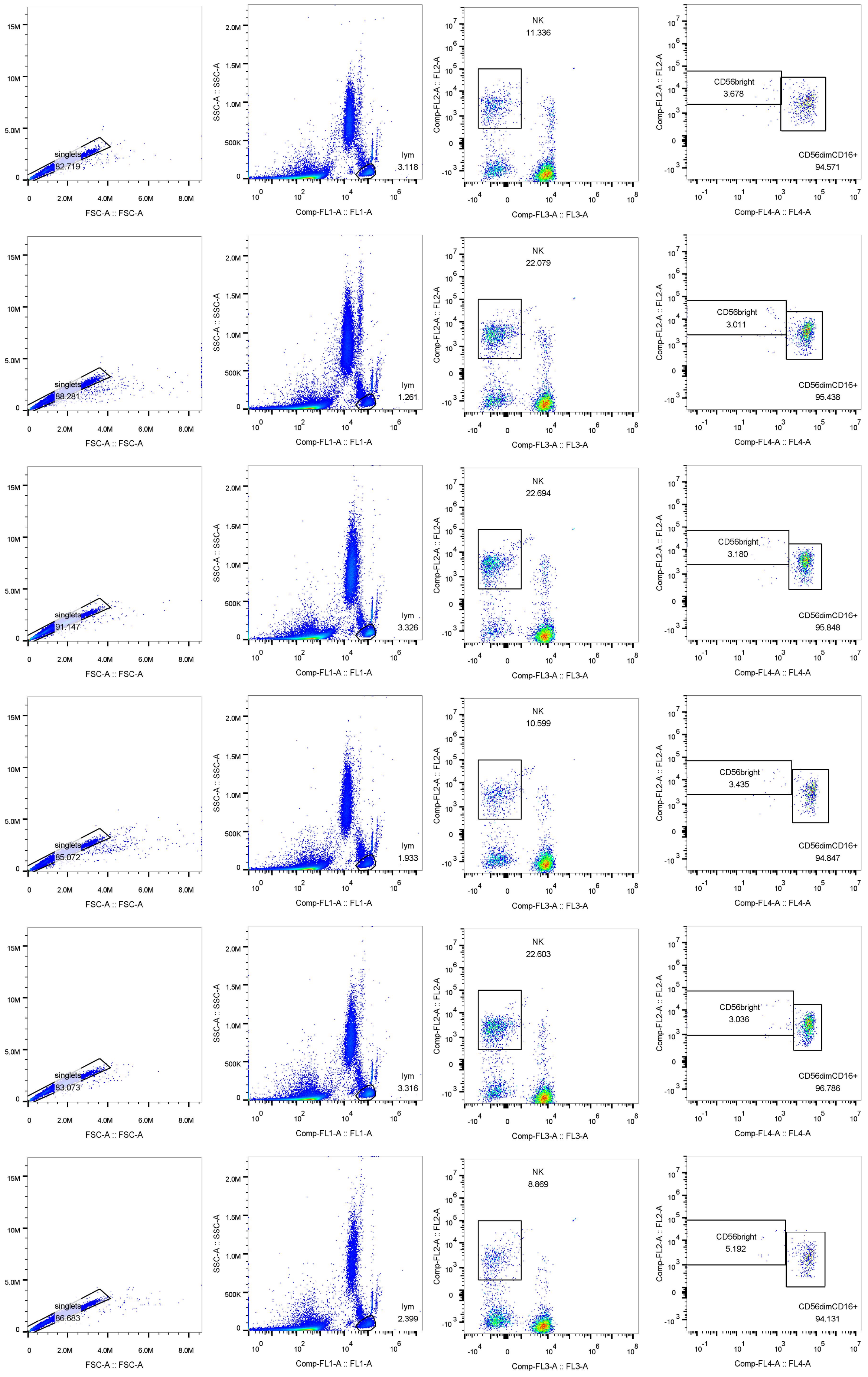

singlets
82.719
FSC-A :: FSC-A
lym
3.118
SSC-A :: SSC-A
Comp-FL1-A :: FL1-A
NK
11.336
Comp-FL2-A :: FL2-A
Comp-FL3-A :: FL3-A
CD56bright
3.678
CD56dimCD16+
94.571
Comp-FL4-A :: FL4-A
singlets
88.281
lym
1.261
NK
22.079
CD56bright
3.011
CD56dimCD16+
95.438
singlets
91.147
lym
3.326
NK
22.694
CD56bright
3.180
CD56dimCD16+
95.848
singlets
85.072
lym
1.933
NK
10.599
CD56bright
3.435
CD56dimCD16+
94.847
singlets
83.073
lym
3.316
NK
22.603
CD56bright
3.036
CD56dimCD16+
96.786
singlets
86.683
lym
2.399
NK
8.869
CD56bright
5.192
CD56dimCD16+
94.131

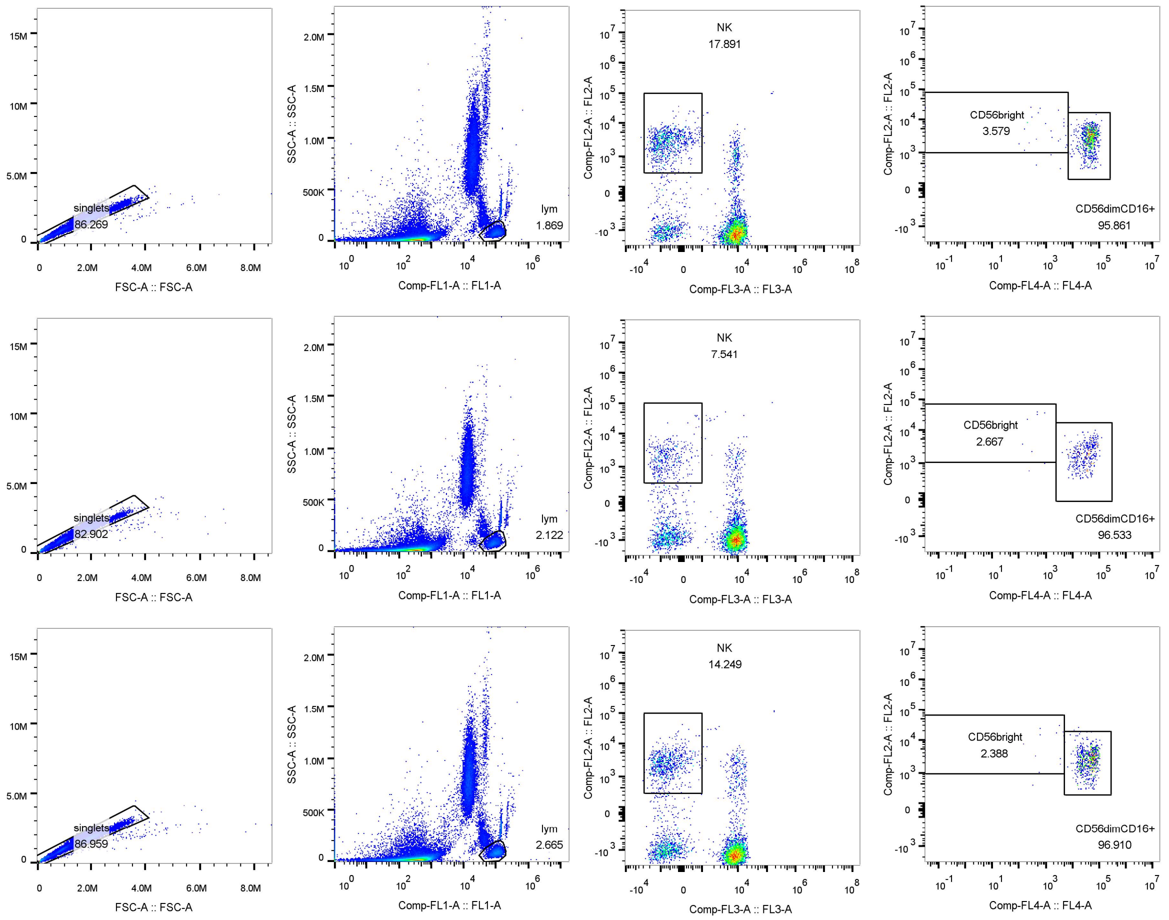

Figure S1. Display of the frequency distribution of CD56dim and CD56bright natural killer cell subsets in the HC group.
