# Supplementary material for: Transcriptomic and proteomic signatures of host NK cells delineate distinct immune states across tuberculosis infection statuses
Source: Front Immunol. 2025 Jun 16;16:1607770. doi: 10.3389/fimmu.2025.1607770 (PMC12206628; doi:10.3389/fimmu.2025.1607770)
Supplement: Supplementary file 2 [file DataSheet2.pdf]

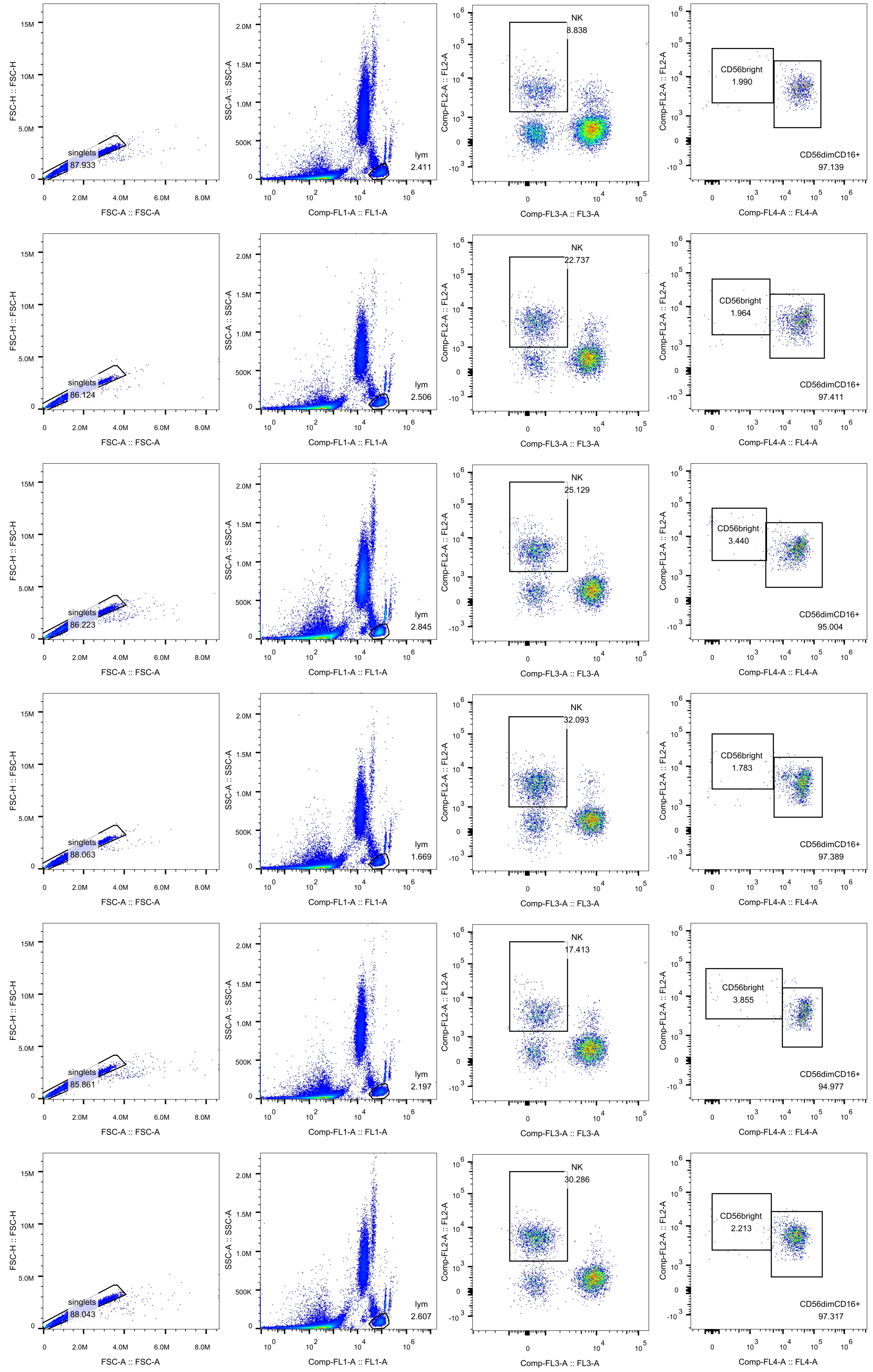
FSC-H :: FSC-H
FSC-A :: FSC-A
singlets
87.933
SSC-A :: SSC-A
Comp-FL1-A :: FL1-A
lym
2.411
Comp-FL2-A :: FL2-A
Comp-FL3-A :: FL3-A
NK
8.838
Comp-FL2-A :: FL2-A
Comp-FL4-A :: FL4-A
CD56bright
1.990
CD56dimCD16+
97.139
singlets
86.124
lym
2.506
NK
22.737
CD56bright
1.964
CD56dimCD16+
97.411
singlets
86.223
lym
2.845
NK
25.129
CD56bright
3.440
CD56dimCD16+
95.004
singlets
88.063
lym
1.669
NK
32.093
CD56bright
1.783
CD56dimCD16+
97.389
singlets
85.861
lym
2.197
NK
17.413
CD56bright
3.855
CD56dimCD16+
94.977
singlets
88.043
lym
2.607
NK
30.286
CD56bright
2.213
CD56dimCD16+
97.317

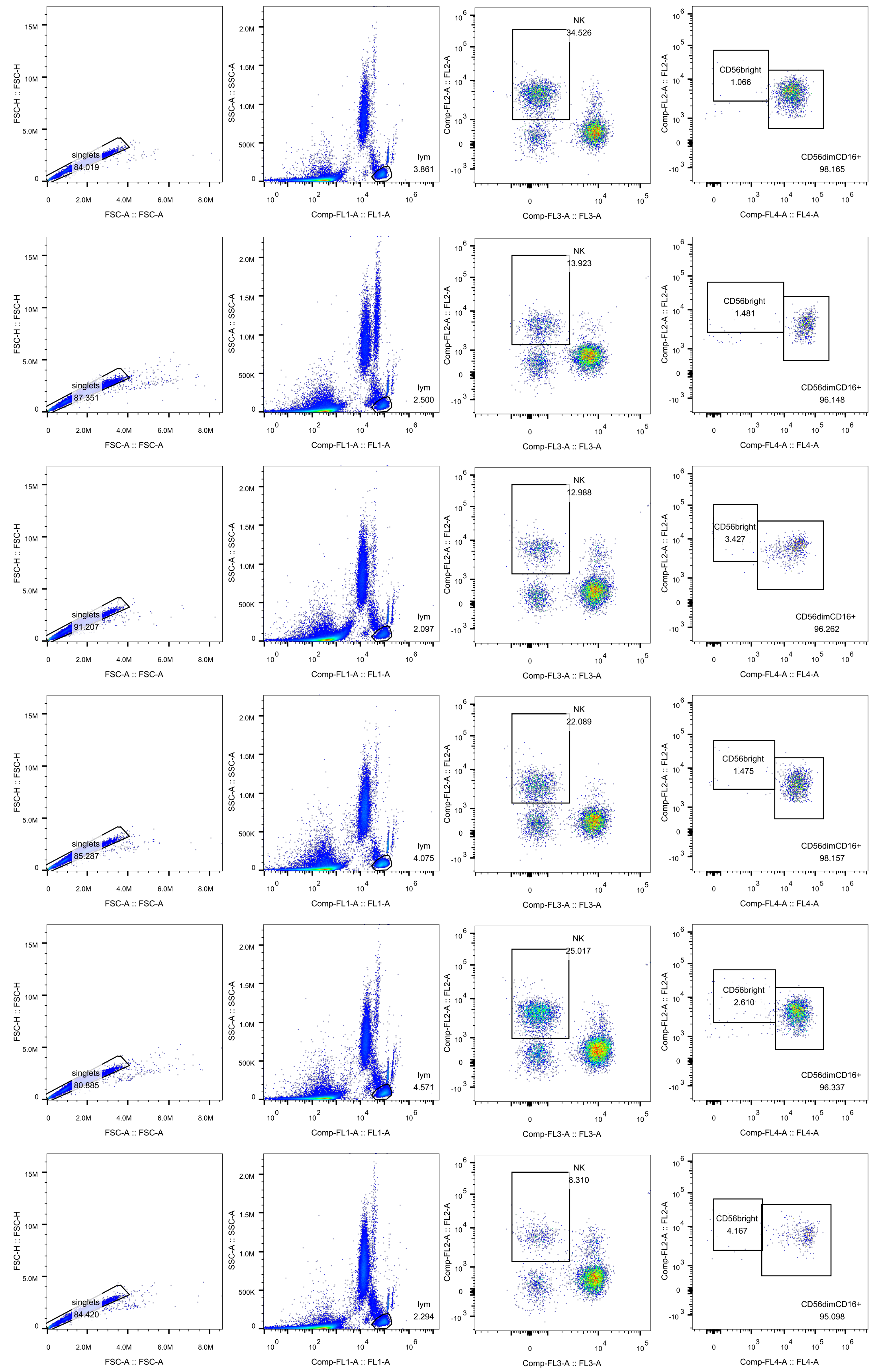

singlets
84.019
lym
3.861
NK
34.526
CD56bright
1.066
CD56dimCD16+
98.165
singlets
87.351
lym
2.500
NK
13.923
CD56bright
1.481
CD56dimCD16+
96.148
singlets
91.207
lym
2.097
NK
12.988
CD56bright
3.427
CD56dimCD16+
96.262
singlets
85.287
lym
4.075
NK
22.089
CD56bright
1.475
CD56dimCD16+
98.157
singlets
80.885
lym
4.571
NK
25.017
CD56bright
2.610
CD56dimCD16+
96.337
singlets
84.420
lym
2.294
NK
8.310
CD56bright
4.167
CD56dimCD16+
95.098
FSC-H :: FSC-H
FSC-A :: FSC-A
SSC-A :: SSC-A
Comp-FL1-A :: FL1-A
Comp-FL2-A :: FL2-A
Comp-FL3-A :: FL3-A
Comp-FL4-A :: FL4-A

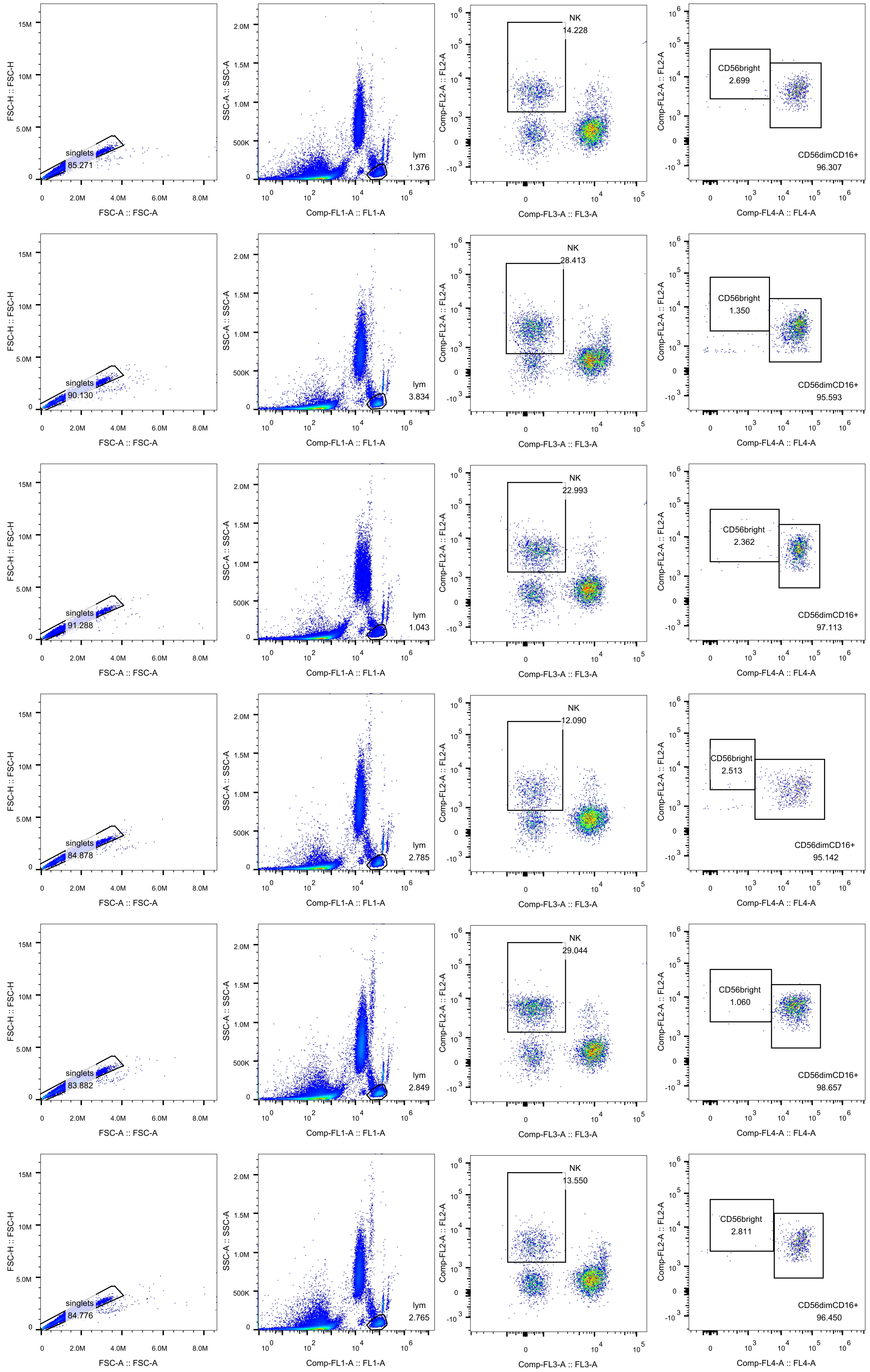
FSC-H :: FSC-H
FSC-A :: FSC-A
SSC-A :: SSC-A
Comp-FL1-A :: FL1-A
Comp-FL2-A :: FL2-A
Comp-FL3-A :: FL3-A
Comp-FL4-A :: FL4-A
singlets
85.271
lym
1.376
NK
14.228
CD56bright
2.699
CD56dimCD16+
96.307
singlets
90.130
lym
3.834
NK
28.413
CD56bright
1.350
CD56dimCD16+
95.593
singlets
91.288
lym
1.043
NK
22.993
CD56bright
2.362
CD56dimCD16+
97.113
singlets
84.878
lym
2.785
NK
12.090
CD56bright
2.513
CD56dimCD16+
95.142
singlets
83.882
lym
2.849
NK
29.044
CD56bright
1.060
CD56dimCD16+
98.657
singlets
84.776
lym
2.765
NK
13.550
CD56bright
2.811
CD56dimCD16+
96.450

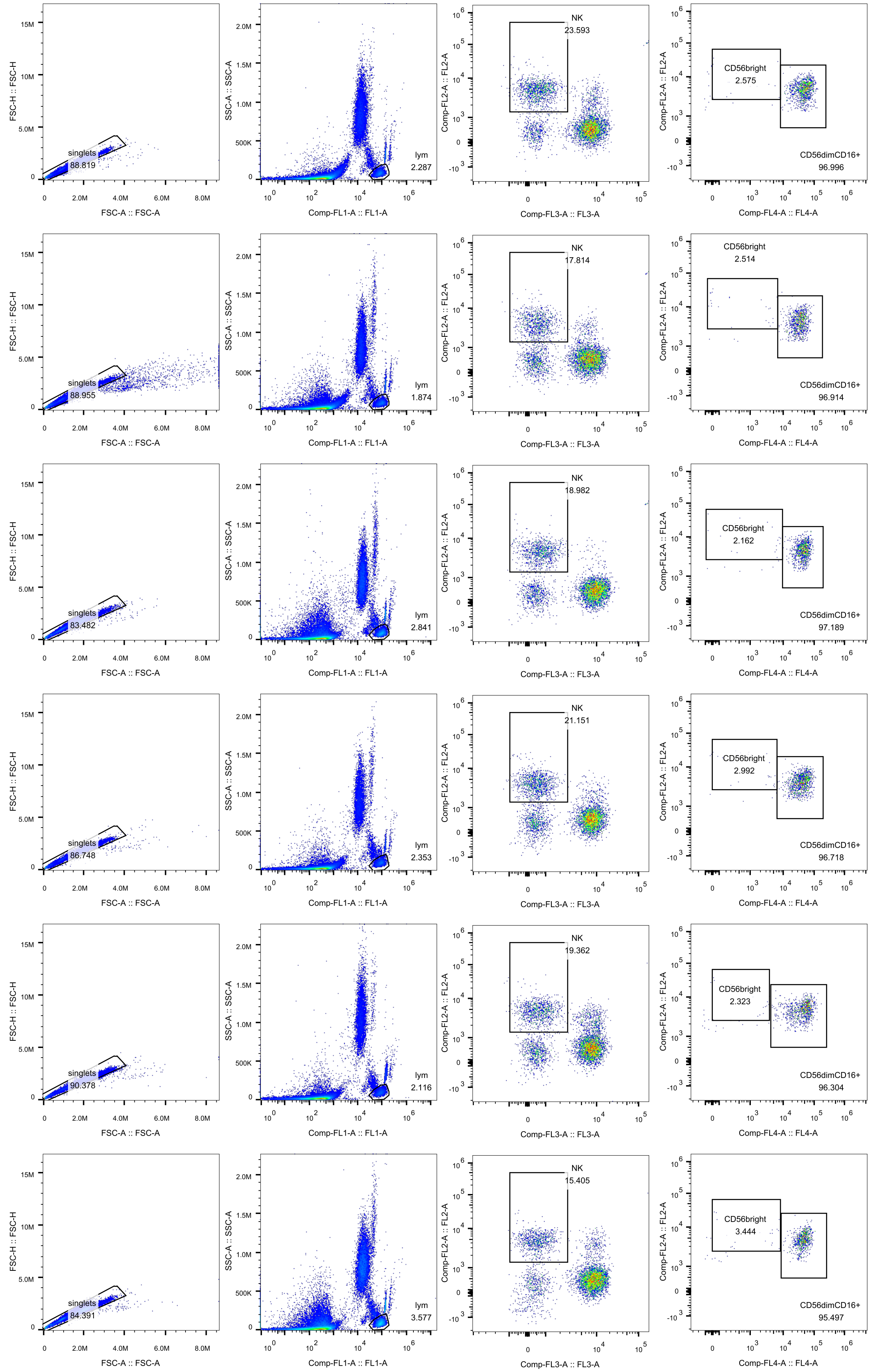

FSC-H :: FSC-H
FSC-A :: FSC-A
SSC-A :: SSC-A
Comp-FL1-A :: FL1-A
Comp-FL2-A :: FL2-A
Comp-FL3-A :: FL3-A
Comp-FL4-A :: FL4-A
singlets
88.819
lym
2.287
NK
23.593
CD56bright
2.575
CD56dimCD16+
96.996
singlets
88.955
lym
1.874
NK
17.814
CD56bright
2.514
CD56dimCD16+
96.914
singlets
83.482
lym
2.841
NK
18.982
CD56bright
2.162
CD56dimCD16+
97.189
singlets
86.748
lym
2.353
NK
21.151
CD56bright
2.992
CD56dimCD16+
96.718
singlets
90.378
lym
2.116
NK
19.362
CD56bright
2.323
CD56dimCD16+
96.304
singlets
84.391
lym
3.577
NK
15.405
CD56bright
3.444
CD56dimCD16+
95.497

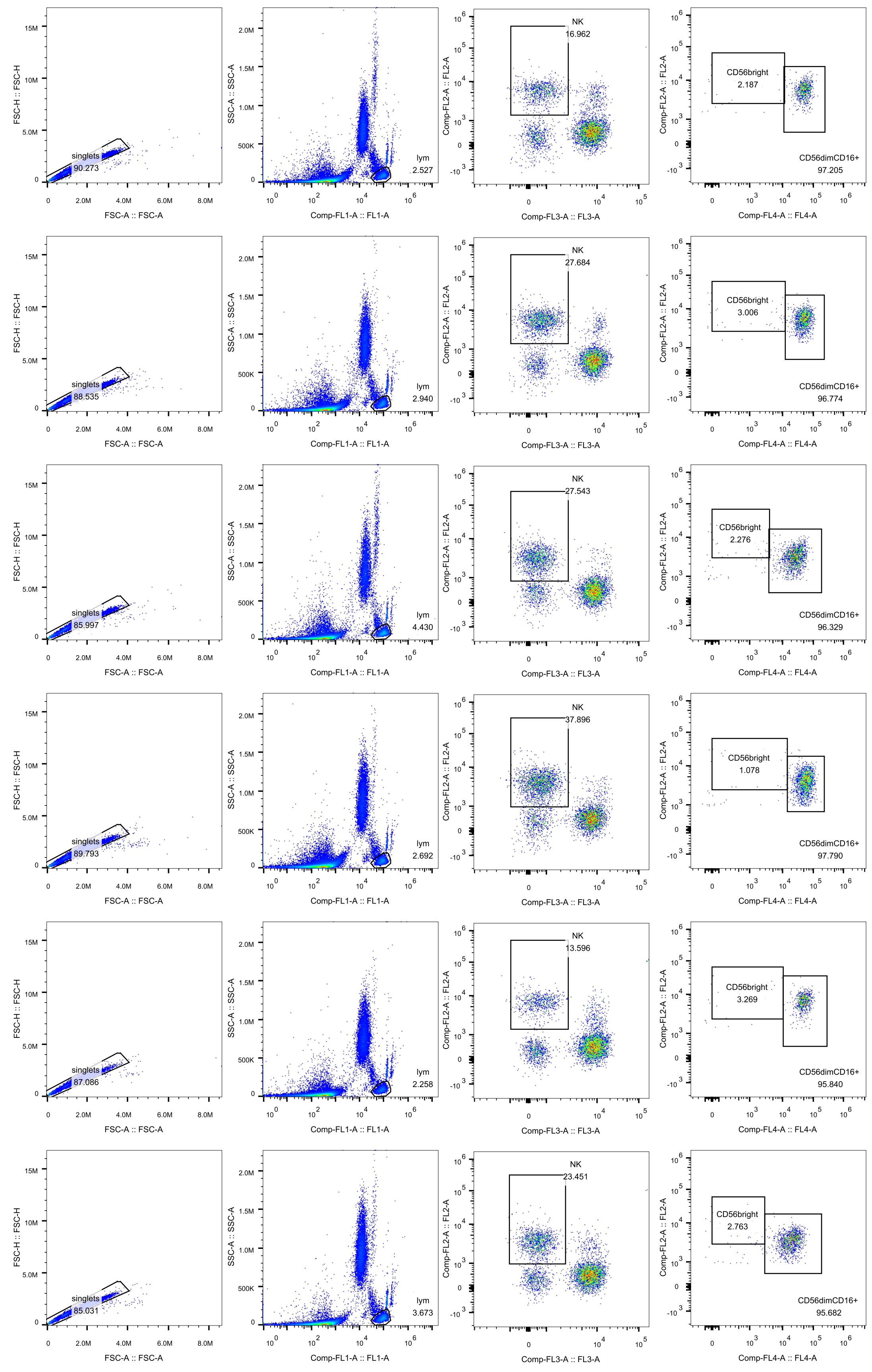
FSC-H :: FSC-H
FSC-A :: FSC-A
SSC-A :: SSC-A
Comp-FL1-A :: FL1-A
Comp-FL2-A :: FL2-A
Comp-FL3-A :: FL3-A
Comp-FL4-A :: FL4-A
singlets 90.273
lym 2.527
NK 16.962
CD56bright 2.187
CD56dimCD16+ 97.205
singlets 88.535
lym 2.940
NK 27.684
CD56bright 3.006
CD56dimCD16+ 96.774
singlets 85.997
lym 4.430
NK 27.543
CD56bright 2.276
CD56dimCD16+ 96.329
singlets 89.793
lym 2.692
NK 37.896
CD56bright 1.078
CD56dimCD16+ 97.790
singlets 87.086
lym 2.258
NK 13.596
CD56bright 3.269
CD56dimCD16+ 95.840
singlets 85.031
lym 3.673
NK 23.451
CD56bright 2.763
CD56dimCD16+ 95.682

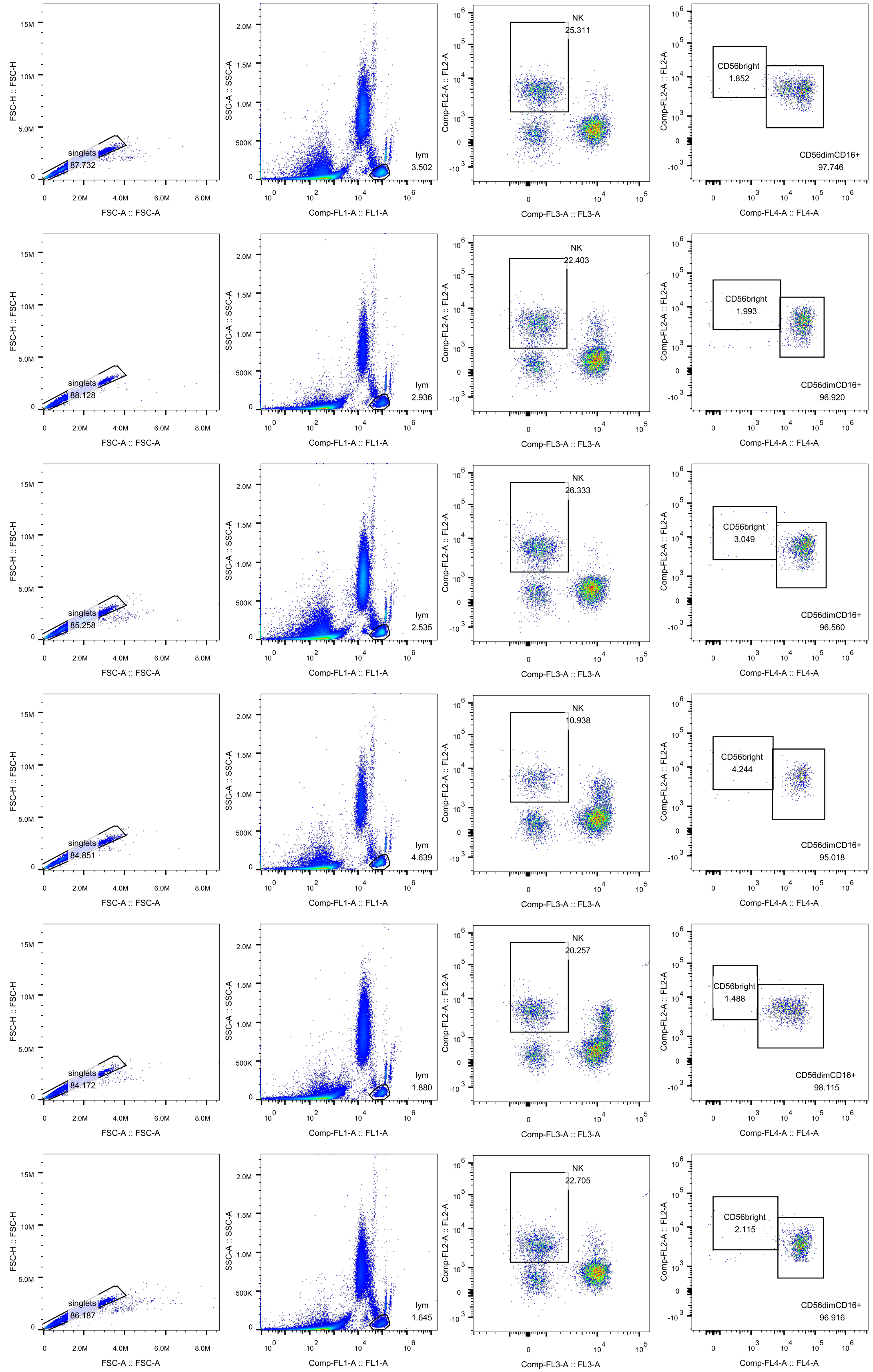
FSC-H :: FSC-H
FSC-A :: FSC-A
SSC-A :: SSC-A
Comp-FL1-A :: FL1-A
Comp-FL2-A :: FL2-A
Comp-FL3-A :: FL3-A
Comp-FL4-A :: FL4-A
singlets
87.732
lym
3.502
NK
25.311
CD56bright
1.852
CD56dimCD16+
97.746
singlets
88.128
lym
2.936
NK
22.403
CD56bright
1.993
CD56dimCD16+
96.920
singlets
85.258
lym
2.535
NK
26.333
CD56bright
3.049
CD56dimCD16+
96.560
singlets
84.851
lym
4.639
NK
10.938
CD56bright
4.244
CD56dimCD16+
95.018
singlets
84.172
lym
1.880
NK
20.257
CD56bright
1.488
CD56dimCD16+
98.115
singlets
86.187
lym
1.645
NK
22.705
CD56bright
2.115
CD56dimCD16+
96.916

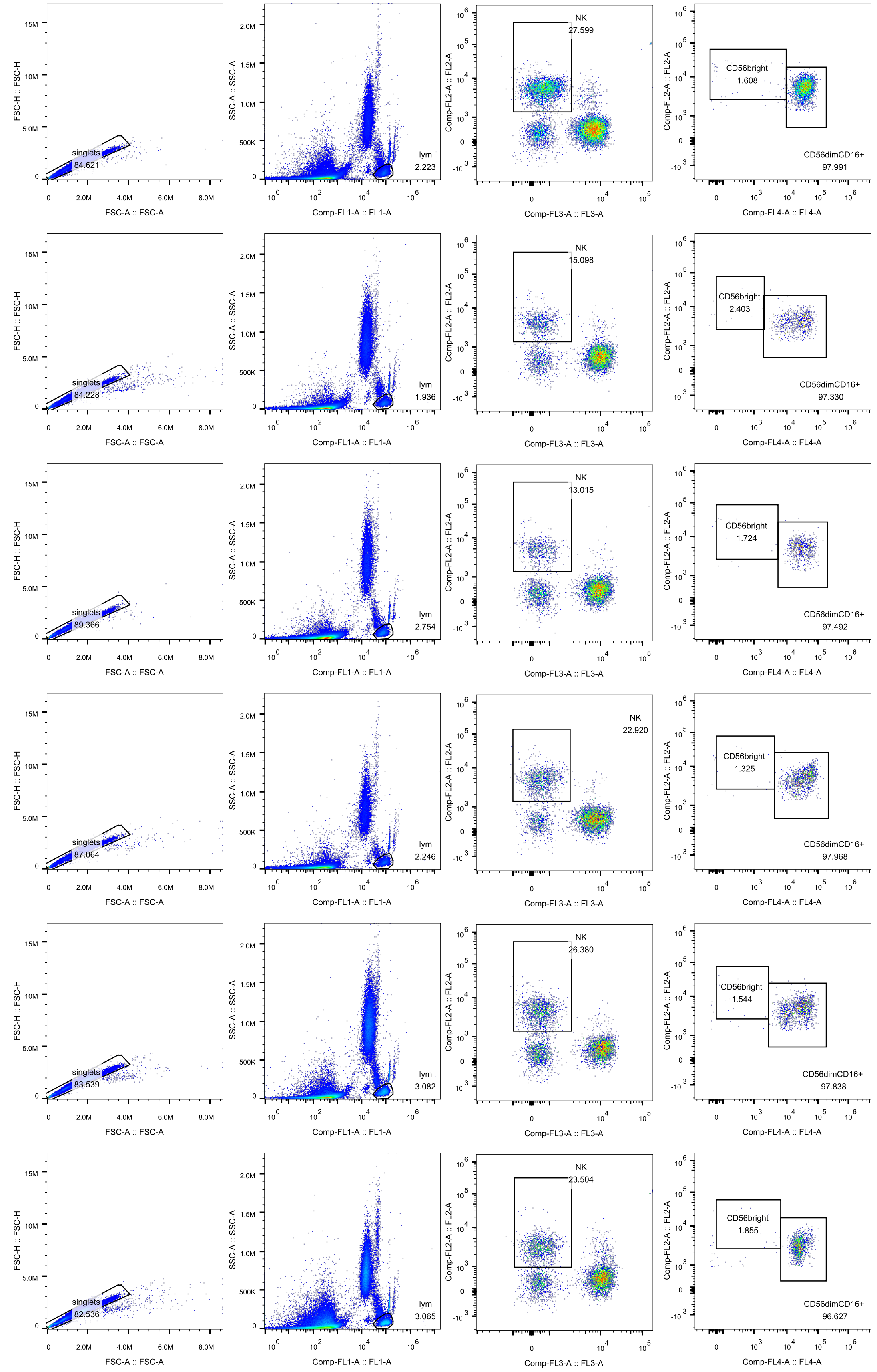
FSC-H :: FSC-H
FSC-A :: FSC-A
SSC-A :: SSC-A
Comp-FL1-A :: FL1-A
Comp-FL2-A :: FL2-A
Comp-FL3-A :: FL3-A
Comp-FL4-A :: FL4-A
singlets 84.621
lym 2.223
NK 27.599
CD56bright 1.608
CD56dimCD16+ 97.991
singlets 84.228
lym 1.936
NK 15.098
CD56bright 2.403
CD56dimCD16+ 97.330
singlets 89.366
lym 2.754
NK 13.015
CD56bright 1.724
CD56dimCD16+ 97.492
singlets 87.064
lym 2.246
NK 22.920
CD56bright 1.325
CD56dimCD16+ 97.968
singlets 83.539
lym 3.082
NK 26.380
CD56bright 1.544
CD56dimCD16+ 97.838
singlets 82.536
lym 3.065
NK 23.504
CD56bright 1.855
CD56dimCD16+ 96.627

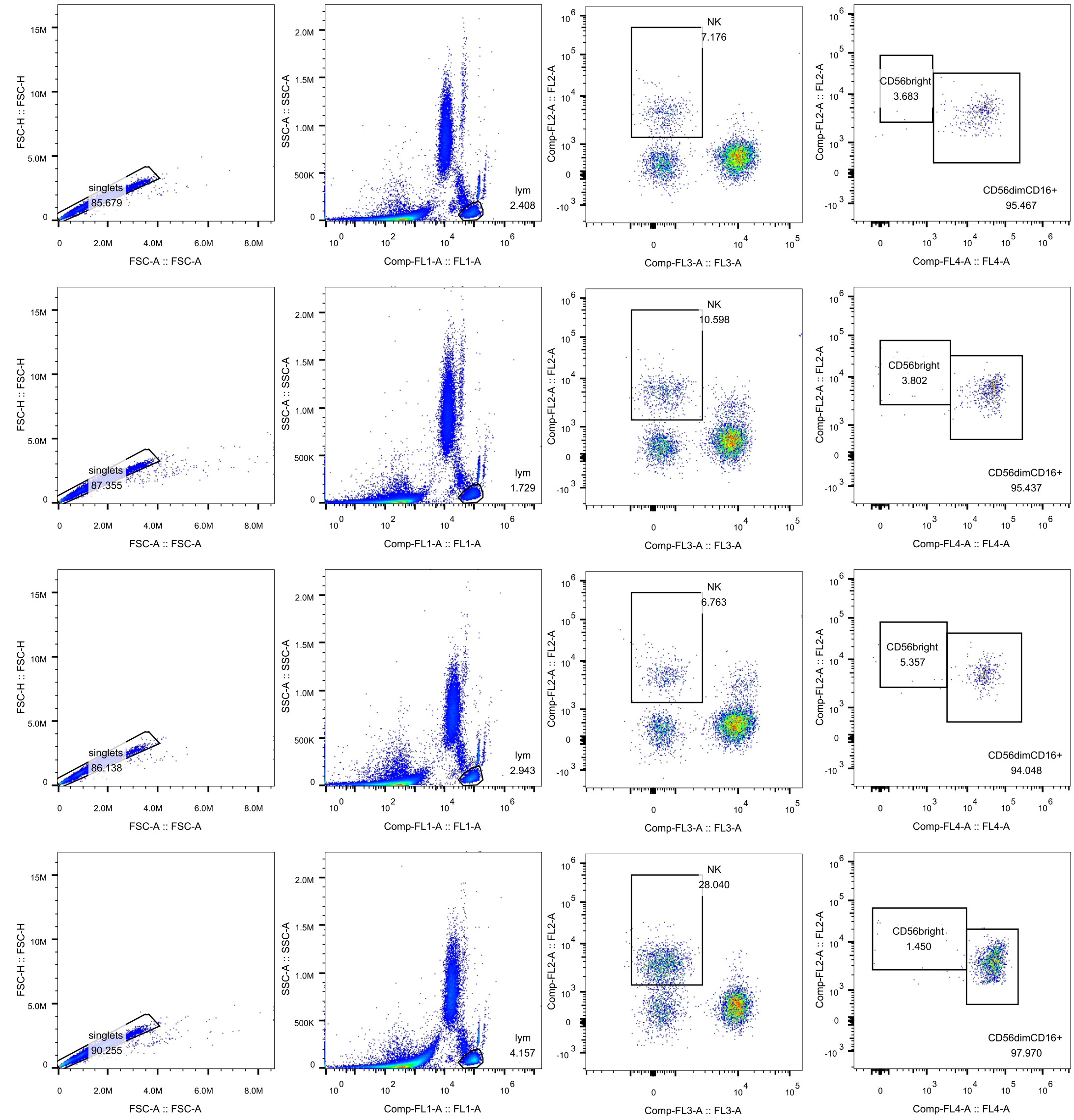

Figure S2. Display of the frequency distribution of CD56dim and CD56bright natural killer cell subsets in the LTBI group.
